# Salinity induces discontinuous protoxylem via a DELLA‐dependent mechanism promoting salt tolerance in Arabidopsis seedlings

**DOI:** 10.1111/nph.18339

**Published:** 2022-07-12

**Authors:** Frauke Augstein, Annelie Carlsbecker

**Affiliations:** ^1^ Department of Organismal Biology, Physiological Botany, and Linnean Centre for Plant Biology Uppsala University Ullsv. 24E SE‐756 51 Uppsala Sweden

**Keywords:** Arabidopsis, DELLA, gibberellins, root, salt, seedling, xylem

## Abstract

- Salinity is detrimental to plants and developmental adjustments limiting salt uptake and transport is therefore important for acclimation to high salt. These parameters may be influenced by xylem morphology, however how plant root xylem development is affected by salt stress remains unclear.
- Using molecular and genetic techniques and detailed phenotypic analyses, we demonstrate that salt causes distinct effects on Arabidopsis seedling root xylem and reveal underlying molecular mechanisms.
- Salinity causes intermittent inhibition of protoxylem cell differentiation, generating protoxylem gaps, in Arabidopsis and several other eudicot seedlings. The extent of protoxylem gaps in seedlings positively correlates with salt tolerance. Reduced gibberellin signalling is required for protoxylem gap formation. Mutant analyses reveal that the xylem differentiation regulator VASCULAR RELATED NAC DOMAIN 6 (VND6), along with secondary cell wall producing and cell wall modifying enzymes, including EXPANSIN A1 (EXP1), are involved in protoxylem gap formation, in a DELLA‐dependent manner.
- Salt stress is likely to reduce levels of bioactive gibberellins, stabilising DELLAs, which in turn activates multiple factors modifying protoxylem differentiation. Salt stress impacts seedling survival and formation of protoxylem gaps may be a measure to enhance salt tolerance.

## Introduction

Survival of plant seedlings is affected by many environmental parameters such as available water and soil salinity. Salt has a negative impact on the plant both through its osmotic effect, which may result in reduced ability for water uptake, and because of ion toxicity (Munns & Tester, 2008). It affects many important processes including photosynthesis, respiration, ion uptake and membrane integrity (West *et al*., 2004; Tavakkoli *et al*., 2011; Talei *et al*., 2012; Mansour, 2013; Awlia *et al*., 2021; Zhao *et al*., 2021). Salt stress tolerance is expected to involve avoidance mechanisms and reduced uptake and transport of salt ions (Møller *et al*., 2009). The initial response to saline conditions is a growth arrest of both primary and lateral roots followed by a temporally dynamic acclimation process in which growth is restored and salt tolerance mechanisms activated (Geng *et al*., 2013). Therefore, it is conceivable that salt stress also affects the development of the water transporting tissue, the xylem, as that would impact salt uptake. However, how salt affects xylem development is not well known.

**Fig. 1:**
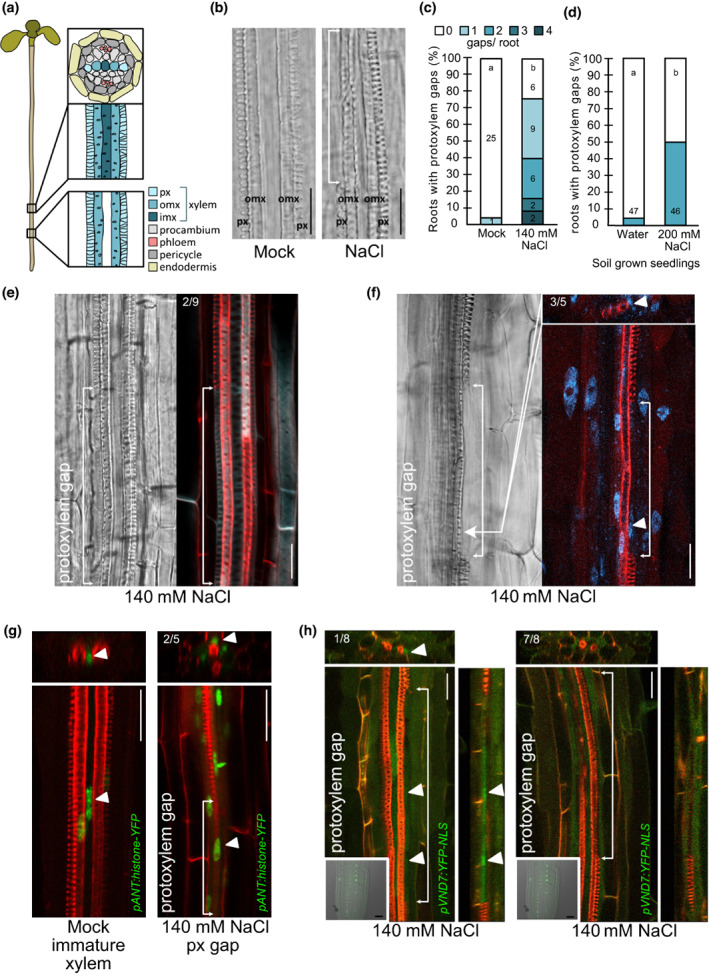
Protoxylem gaps are formed in response to salt. All images depict roots of 6‐d‐old Arabidopsis seedlings grown for 3 d on 140 mM NaCl or under mock conditions. (a) Cartoon of Arabidopsis seedling root xylem in longitudinal view and stele + endodermis in cross‐section. imx, inner metaxylem; omx, outer metaxylem; px, protoxylem. (b) Differential interference contrast (DIC) images of root xylem. White arrows indicate a protoxylem gap. Bar = 50 μm. (c) Quantification of roots exhibiting different amounts of protoxylem gaps. Number of roots showing the phenotype are indicated in the graph; letters indicate statistical significance considering no protoxylem gaps and any amount of protoxylem gaps with multiple Fisher's exact test and Benjamini–Hochberg (BH) correction, *P* < 0.05. (d) Quantification of soil‐grown roots exhibiting protoxylem gaps. Number of analysed roots (*n*) is indicated on the bars; letters indicate statistical significance with multiple Fisher's exact test and BH correction, *P* < 0.05. (e–h) DIC and confocal micrographs of root xylem. White arrows indicate protoxylem gaps. Arrowheads point at nuclei in protoxylem gap cells. Numbers indicate fraction of analysed gaps that showed (e) cellulose secondary cell wall (SCW) pattern, (f) nuclear signal within the gap, (g) *pANT:histone‐YFP* expression within the gap, (h) *pVND7:YFP‐NLS*. Turquoise, Calcofluor White staining cellulose; red, Basic Fuchsin staining lignin; blue, DAPI staining nucleus; green, *pANT:histone‐YFP* or *pVND7:YFP‐NLS*. Bar = 20 μm.

The xylem harbours vessel strands of hollow cells reinforced with lignified secondary cell walls (SCW). In the Arabidopsis root the xylem forms an axis traversing the stele. The two outer strands of the xylem axis differentiate as protoxylem with annular or helical SCW, whereas metaxylem with pitted SCW occupies the central positions of the axis (Fig. 1a). The diameter and shape of the SCWs are thought to influence hydraulic conductance, and the xylem shape correlates with drought resistance in many different species (Arend & Fromm, 2007; Awad *et al*., 2010; Tang *et al*., 2018; Yu *et al*., 2021). Recently, we and others showed that xylem formation is plastic and responds to water availability. Under conditions of reduced water availability, extra protoxylem strands form, and metaxylem differentiates closer to the root tip (Ramachandran *et al*., 2018, 2021; Bloch *et al*., 2019). The hormone abscisic acid (ABA) mediates these developmental responses by at least two different mechanisms. Firstly, ABA promotes the production of miR165 in the endodermis (Ramachandran *et al*., 2018; Bloch *et al*., 2019). This miRNA moves into the stele to reduce homeodomain leucine zipper class III (HD‐ZIP III) mRNA levels, eventually leading to protoxylem formation in place of metaxylem (Carlsbecker *et al*., 2010; Miyashima *et al*., 2011). Secondly, ABA directly promotes the expression of VASCULAR RELATED NAC DOMAIN (VND) transcription factors within the immature xylem cells, where VND7 promotes protoxylem formation, and VND2 and VND3 metaxylem differentiation closer to the root tip (Ramachandran *et al*., 2021).

Salt stress also triggers ABA signalling, but recent research has in addition highlighted the importance of gibberellin (GA) levels and signalling. Reduced GA levels or signalling may result in salt stress tolerance (Colebrook *et al*., 2014), whereas the absence of the DELLA repressors of GA signalling make Arabidopsis less salt tolerant (Achard *et al*., 2006). In line with these findings, salt stress leads to a reduction in bioactive GA levels, which in turn stabilises DELLAs (Achard *et al*., 2006; Magome *et al*., 2008). Under normal conditions, GAs affect xylem lignification in both primary and secondary development in several different species (Eriksson *et al*., 2000; Mauriat & Moritz, 2009; Gou *et al*., 2011; Ragni *et al*., 2011; Guo *et al*., 2015; Wang *et al*., 2017; Singh *et al*., 2019), GAs promote xylem formation in secondary development and are important for fibre development (Mauriat & Moritz, 2009; Ragni *et al*., 2011; Felipo‐Benavent *et al*., 2018). Furthermore, DELLAs are implicated in the regulation of cell wall synthesis and remodelling in Arabidopsis (Locascio *et al*., 2013; Felipo‐Benavent *et al*., 2018).

Here, we analyse the effect of salt stress on Arabidopsis seedling root xylem development. We show that salt stress results in discontinuous differentiation of the protoxylem strands generating protoxylem gaps. The capacity to form protoxylem gaps correlates with salt tolerance, indicating that gaps promote survival under high salinity. We also show that the formation of protoxylem gaps under salt stress requires DELLA‐mediated repression of GA signalling. Under salt stress, DELLAs promote the expression of *VND6* and genes encoding SCW enzymes, as well as genes encoding multiple cell wall modifying enzymes including alpha‐expansins such as EXP1, also called EXPA1. The loss of *VND6* or *EXP1* consequently results in less protoxylem gaps forming under salt stress.

## Materials and Methods

### Plant material and growth conditions

Seeds were surface sterilised using 70% ethanol for 20 min and 95% ethanol for 2 min, and then rinsed four times for 2 min in sterile water. The seeds were plated on half‐strength Murashige and Skoog (½MS) medium (Murashige & Skoog, 1962), pH 5.7–5.8, with 0.05% MES monohydrate and 1% Bactoagar, and stratified for 48 h at 4°C. Plants were grown in long day conditions, with cycles of 16 h light at 110 μE light intensity and 8 h darkness. Temperatures were 22°C (light), 20°C (darkness). For all experiments, plants were grown vertically on 25 mm pore Sefar Nitex 03–25/19 mesh, and transferred to new plates by transferring the mesh supporting the plants for minimal disturbance. For GA_3_, a stock solution in 99.9% EtOH was prepared; for GA_4+7_ and paclobutrazol (PAC) stock solutions were prepared in dimethyl sulphoxide (DMSO). For plates with NaCl, a 3 M stock solution was used and diluted in the medium to the indicated concentration. Mannitol was added directly into the medium after autoclaving. For Arabidopsis xylem phenotyping experiments, 3‐d‐old seedlings were transferred to treatment plates (including NaCl, mannitol, gibberellin and ABA) for 3 d. For tolerance assays, 3‐d‐old plants were left on NaCl‐plates for 4–7 d, as indicated. For RNA‐sequencing analysis, 5‐d‐old seedlings were used and exposed to salt for the indicated time. All material was collected at the same time in the afternoon, to avoid circadian clock effects. For phenotyping of *Solanum lycopersicum* L. cv Moneymaker, *Beta vulgaris* L. cv Davinci, and *Eutrema salsugineum* (Pall.) Al‐Shehbaz & Warwick seedlings were grown until roots reached *c*. 1 cm in length before transfer to salt for 3 d. For treatment on soil, seeds were sterilised, stratified and then sown on soil. Here, 3‐d‐old seedlings were watered with 200 mM NaCl solution once per day during a 3‐d period. Seedlings were then removed from the soil, washed and mounted in chloral hydrate (see below) for xylem morphology analysis. Detailed information about all genotypes can be found in Supporting information Methods S1.

### Root length measurements

Root lengths were measured using Fiji/ImageJ software.

### Xylem phenotype analysis

For analysis of xylem morphology, roots were mounted in chloralhydrate solution, 8 : 3 : 1 chloralhydrate : water : glycerol (w/v/v), and visualised, as previously described, using a Zeiss Axioscope A1 microscope at ×40 magnification with differential interference contrast (DIC) optics (Ramachandran *et al*., 2018, 2021). Frequency of plants exhibiting the protoxylem gap phenotype was scored, and the number of gaps per root was counted.

### Salt tolerance assay

Colouring of cotyledons of seedlings grown on high salt concentrations was determined after 4 or 7 d, as indicated. Plants exhibiting white, pale green or green cotyledons were counted separately. From the fraction of each category a survival score was calculated multiplying white with 1, pale with 3 and green with 5 divided by the sum, following (Gibbs *et al*., 2011). Data from one experiment with 3–5 replicates per genotype‐treatment combination is presented. The experiment was repeated two or three times (Table S1).

### Confocal analysis

For parallel staining of Basic Fuchsin and Calcofluor White or DAPI, we followed a modified fixation protocol from Ursache *et al*. (2018). Here, 6‐d old seedlings were fixed with 4% PFA in 1× PBS, for 1 h for Basic Fuchsin and Calcofluor White, 10–15 min for Basic Fuchsin and DAPI staining or 10–15 min for Basic Fuchsin stain and transcriptional reporter lines. This was followed by twice washing for 1 min with 1× PBS, and then clearing overnight with ClearSee (10% xylitol, 15% sodium deoxycholate and 25% urea in water). For the Basic Fuchsin stain, seedlings were then stained with 0.2% Basic Fuchsin (in ClearSee) overnight, and washed with ClearSee twice. For Calcofluor White staining, seedlings were stained with 0.1% Calcofluor White for 30 min, and washed with ClearSee for 30 min. For visualisation with confocal microscopy, roots were mounted directly in ClearSee. For DAPI staining, seedlings were mounted in 0.4 μl of 5 mg ml^−1^ DAPI solution in 10 ml H_2_O and visualised directly. For *pRGA:GFP‐RGA* reporter analysis, roots were mounted in 40 mM propidium iodide (PI) solution between two coverslips and imaged immediately.

Confocal micrographs were captured using Zeiss LSM780 inverted Axio Observer microscope with supersensitive GaAsP detectors or an LSM800 inverted confocal microscope. For Calcofluor White, a 405 nm laser was used for excitation and emission and wavelengths at 410–475 nm were captured. For Basic Fuchsin images, wavelengths were 561 nm excitation and 600–700 nm emission. For DAPI images, wavelengths were 405 nm excitation and 410–511 emission. For reporter lines expressing GFP and stained with PI, 561 nm excitation and 650–700 nm emission were used for PI and 488 nm excitation and 410–523 nm emission for GFP. For reporter lines expressing YFP, 514 nm excitation and 518–544 emission were used. For quantification of fluorescence intensity all imaging parameters were kept the same when imaging mock or treated roots. Fluorescence intensity of *pRGA:GFP‐RGA* in the provascular cells of the meristem was quantified using CellSeT (Pound *et al*., 2012). Fluorescence intensity of *pRGA:GFP‐RGA* in the vascular cells at the transition zone was measured using imagej software (Schindelin *et al*., 2012). From a *z*‐stack the plane that transverse the centre of the stele was selected, the vascular tissue was marked and the intensity values were extracted using the plot profile function. From all values the average was calculated. Selected pictures represent the average of all biological replicates.

### RNA‐sequencing analysis

Here, 5‐d‐old Arabidopsis seedlings of Landsberg *erecta* (L*er*; wild‐type) and *gai‐t6 rga‐t2 rgl1‐1 rgl2‐1 rgl3‐4* (*della5x*), were grown on 140 mM NaCl or under mock conditions for 1 or 8 h. For the 8 h timepoint, *ga4* and *gai* were grown in parallel. Three biological replicates, each consisting of 50–100 seedlings, were collected for each treatment–genotype combination. The lower part of the root (1 cm) was collected directly into RLT buffer (Qiagen) and frozen in liquid nitrogen. RNA was extracted using the RNeasy Plant Mini Kit (Qiagen). RNA concentration was measured using the Qubit BR RNA assay and quality and integrity of the RNA was analysed using the Agilent Bioanalyser 2100 system (Agilent Technologies, Santa Clara, CA, USA). In total, 1000 ng RNA per sample were used for library preparation. Library preparation and sequencing were performed by Novogene (UK) on their Illumina sequencing platform with paired‐end read lengths for the 150 and 250–300 bp cDNA library, resulting in 5.9–8.3 G raw data per sample (241.9 G total). fastp was used for quality assessment and adapter trimming (Chen *et al*., 2018). Mapping to the *Arabidopsis thaliana* reference genome (TAIR10) was carried out using Hisat2 (Kim *et al*., 2019). Here, 96% of the total reads were mapped to the Arabidopsis genome, whereby 93% of the total reads were uniquely mapped. Count files were generated using HTSeq‐Count (Anders *et al*., 2015). Differential expression analysis was done using DESeq2 in Bioconductor (Huber *et al*., 2015). For statistical analysis of the effect of NaCl on the different genotypes compared with wild‐type, a DESeq2 model including a combinatorial effect was used (~genotype + genotype : condition). Log_2_ fold changes (FC) were extracted from the pairwise comparison of mock vs treatment for each genotype, while *P*‐values and adjusted *P*‐values were extracted from the comparison between the mutants and wild‐type. The effect of the different genotypes under mock conditions was analysed in a separate differential expression analysis and all values were extracted from the pairwise comparison of wild‐type vs mutant. A cut‐off > 0.5 or < −0.5 log_2_FC was applied.

Gene Ontology (GO) term analysis was performed using the Panther classification system (Mi *et al*., 2019, 2021) using *Arabidopsis thaliana* as a reference and the GO annotation dataset ‘biological process complete’. Fisher's exact test was selected as test type and Bonferroni correction for multiple testing was performed. Gene Ontology term clustering was performed using Revigo and *P*‐values (Supek *et al*., 2011). *Arabidopsis thaliana* was used as a reference, obsolete GO terms were removed and SimRel was used as semantic similarity measure.

### Statistical analysis

For categorical data, Fisher's exact test using the *fisher.multcomp()* function of the rvaidememoire package (Hervé, 2021) in R was performed and *P‐*values < 0.05 were considered significant. The *P*‐values were corrected for multiple testing using the Benjamini and Hochberg correction (Benjamini & Hochberg, 1995). For other data, two‐way ANOVA, using the *aov()* function in R combined with a Tukey post hoc test or *t*‐tests using the *t.test()* function were used as indicated. Statistical tests and significance thresholds are mentioned in figure legends. Number of roots analysed are mentioned in the corresponding figures.

## Results

### Salt stress inhibits local protoxylem differentiation causing discontinuous xylem strands

To assess how salt stress affects seedling root xylem development we grew 3‐d‐old Arabidopsis Col‐0 seedlings on 140 mM NaCl for 3 d and then analysed primary root growth and xylem morphology in the now 6‐d‐old plants. This concentration is high but nonlethal for 5‐d‐old plants (Dinneny *et al*., 2008). Consistent with previous findings, in which transfer to salt resulted in an initial growth inhibition relieved after some time of acclimation (Geng *et al*., 2013), root growth on salt was substantially reduced (Fig. S1b). We found that the predominant effect on xylem morphology was an appearance of discontinuous protoxylem, seen as protoxylem gaps spread along the part of the xylem, which had differentiated during growth on high salt (Figs 1b, S1a). Therefore, the protoxylem gaps were likely to be not an effect of an initial root growth inhibition upon transfer to high salt concentrations, but rather an effect of the salt stress itself. In 75% of the seedlings analysed, up to four protoxylem gaps on either one or both xylem strands were induced, with an average of 1.4 gaps per root (Fig. 1c). Growth for 3 d on concentrations ranging from 80 to 140 mM of NaCl revealed a concentration dependence in the frequency of plants displaying protoxylem gaps (Fig. S1d). Growth on 280 mM mannitol, iso‐osmolaric to 140 mM NaCl, only occasionally resulted in protoxylem gap formation (Fig. S1d) suggesting that xylem gap formation is mainly linked to ionic stress rather than the osmotic stress. Formation of protoxylem gaps was also observed in the primary roots of older, 14‐d‐old plants grown for 3 d on 140 mM salt (Fig. S1c) and in soil‐grown 3‐d‐old seedlings watered with 200 mM NaCl solution once a day for 3 d and then rinsed and analysed (Fig. 1d).

While the formation of xylem gaps was consistently observed in response to salt stress, it is possible that this is merely a symptom of the toxicity of the salt ions in combination with the osmotic stress causing collapsed cells rather than a developmentally controlled response suppressing local xylem differentiation. To distinguish between these possibilities, we analysed xylem strands double stained with Basic Fuchsin and Calcofluor White to visualise lignin and cellulose (Ursache *et al*., 2018). This revealed that while lignin was absent in the gaps, cellulose was detected. In two of nine gaps we observed cellulose with SCW patterns (Fig. 1e) reminiscent of the fully lignified neighbouring cells, while other gaps displayed a thin cell wall, indicating that these cells only had a primary cell wall. This suggests that salt impacted on SCW formation and lignin deposition. Following SCW formation a xylem cell would undergo programmed cell death (PCD) to form a hollow tube for water transport (Schuetz *et al*., 2013). To analyse if the gap cells maintained a nucleus we fixed and stained salt‐grown roots with DAPI for DNA and Basic Fuchsin for lignin. In the lignified xylem cells surrounding a gap we could not detect DAPI staining, indicating that these cells had undergone PCD. However, in three of five nonlignified xylem gap cells, we detected localised DAPI staining indicating an intact nucleus (Fig. 1f). Furthermore, *pANT:histone‐YFP*, a reporter for *AINTEGUMENTA* active in procambium, immature xylem cells and vascular cambium (Randall *et al*., 2015), was expressed in two out of five of the salt‐induced gap cells, suggesting that these cells displayed procambial or immature xylem cell identity (Fig. 1g). In one out of eight protoxylem gaps, we observed the expression of *pVND7:YFP‐NLS* (Kubo *et al*., 2005), indicating that some protoxylem gap cells had protoxylem identity (Fig. 1h). As certain xylem gap cells apparently were living, we tested if gap cells could resume differentiation if the plants grown on salt were allowed to continue growth on normal medium. After transfer to normal conditions, we observed few gaps in the part of the root grown under normal conditions, indicating that xylem differentiation then became continuous. However, the root section previously grown under high salt conditions exhibited similar amounts of gaps as for those plants kept on salt, suggesting that the gaps could not continue differentiation upon transfer to nonsalt conditions (Fig. S1e). Therefore, our analyses suggest that salt triggers a local nonreversible suppression of xylem cell differentiation.

### Xylem gap formation is not ABA mediated

Extended growth on 140 mM NaCl for 3, 5 or 7 d revealed that the formation of xylem gaps is a relatively early response, followed by the formation of additional protoxylem strands (Fig. S2a,b). Additional protoxylem formation also occurs under water deficiency or after treatment with ABA (Ramachandran *et al*., 2018; Bloch *et al*., 2019). To test if ABA signalling is similarly important for the generation of xylem gaps we made use of the dominant negative *abi1‐1C* mutant, in which ABA signalling is suppressed (Leung *et al*., 1994; Meyer *et al*., 1994). As previously found, *abi1‐1C* reduced the frequency of additional protoxylem formed upon osmotic stress (Fig. S2c,d), and it also partially suppressed the formation of metaxylem closer to the root tip that happens under growth on both salt and on mannitol (Fig. S2e,f; Ramachandran *et al*., 2018, 2021). By contrast, protoxylem gap formation upon growth on salt was not affected by *abi1‐1C* or reduced ABA signalling in the *snrk2.2 snrk2.3* mutant (Fig. S2g,i,j), nor did ABA treatment cause xylem gaps (Fig. S2h). We note that half of the *snrk2.2 snrk2.3* and 10% of *abi1‐1C* seedlings form protoxylem gaps under control conditions (Fig. S2g,i), indicating that ABA signalling is needed for continuous xylem differentiation consistent with our earlier results (Ramachandran *et al*., 2018). Therefore, whereas formation of additional protoxylem and earlier metaxylem differentiation are ABA‐mediated effects also under growth on salt, xylem gap formation appeared not to require ABA signalling.

### Salt stress induces root xylem gaps in several eudicot species

**Fig. 2:**
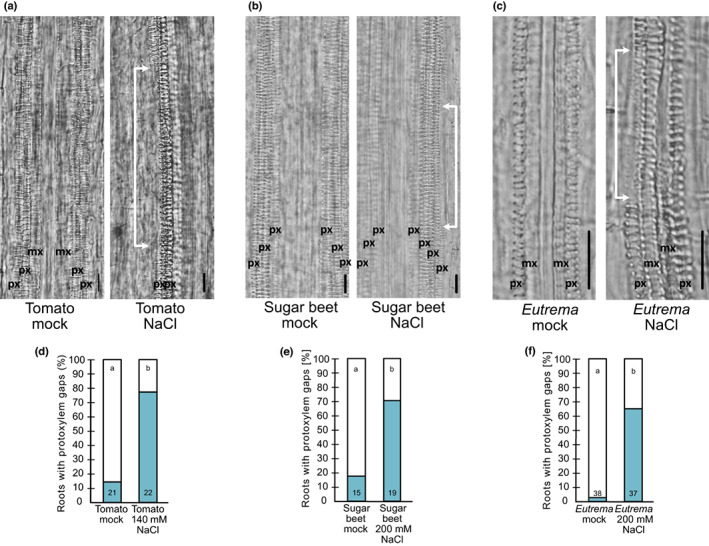
Protoxylem gaps are formed in several eudicot species upon salt. (a) Differential interference contrast (DIC) images of tomato seedling root xylem after growth on NaCl or under mock conditions for 3 d. (b) Sugar beet seedling root xylem after growth on NaCl or under mock conditions for 3 d. (c) *Eutrema* seedling root xylem after growth on NaCl or under mock conditions for 3 d. White arrows indicate protoxylem gaps. mx, metaxylem; px, protoxylem. Bar = 50 μm. (d) Quantification of tomato roots exhibiting protoxylem gaps. (e) Quantification of sugar beet roots exhibiting protoxylem gaps. (f) Quantification of *Eutema* roots exhibiting protoxylem gaps. Numbers in the bars indicated *n*; letters indicate statistical significance with multiple Fisher's exact test and Benjamini–Hochberg (BH) correction, *P* < 0.05.

To elucidate if high salinity‐induced protoxylem gap formation is a general phenomenon in eudicot seedlings, we studied three additional species. We selected tomato (*Solanum lycopersicum*; Solanaceae, subclass Asteridae) that, like Arabidopsis (Brassicaceae, subclass Rosidae), is considered moderately salt sensitive (Munns & Tester, 2008; Sun *et al*., 2010), sugar beet (*Beta vulgaris*; Amaranthaceae, subclass Rosidae) that is salt tolerant (Skorupa *et al*., 2019) and the halophyte *Eutrema salsugineum* (Brassicaceae) (Yang *et al*., 2013). Similar to Arabidopsis, tomato seedlings formed gaps in at least one of the protoxylem strands in most roots analysed when grown on 140 mM NaCl (Fig. 2a,d). For sugar beet and *E. salsugineum* seedlings roots 140 mM NaCl did not significantly enhance gap formation, whereas growth on 200 mM NaCl did (Figs 2b,c,e,f, S3a,b). In *E. salsugineum*, growth on 200 mM salt also induced additional protoxylem strand formation (Fig. S3c). These results indicated that xylem gaps formed upon salt stress in distantly related eudicot species, regardless of whether these were salt sensitive, salt tolerant or even halophytes, if exposed to a high enough salt concentration.

### Gibberellin levels affect protoxylem gap formation

**Fig. 3:**
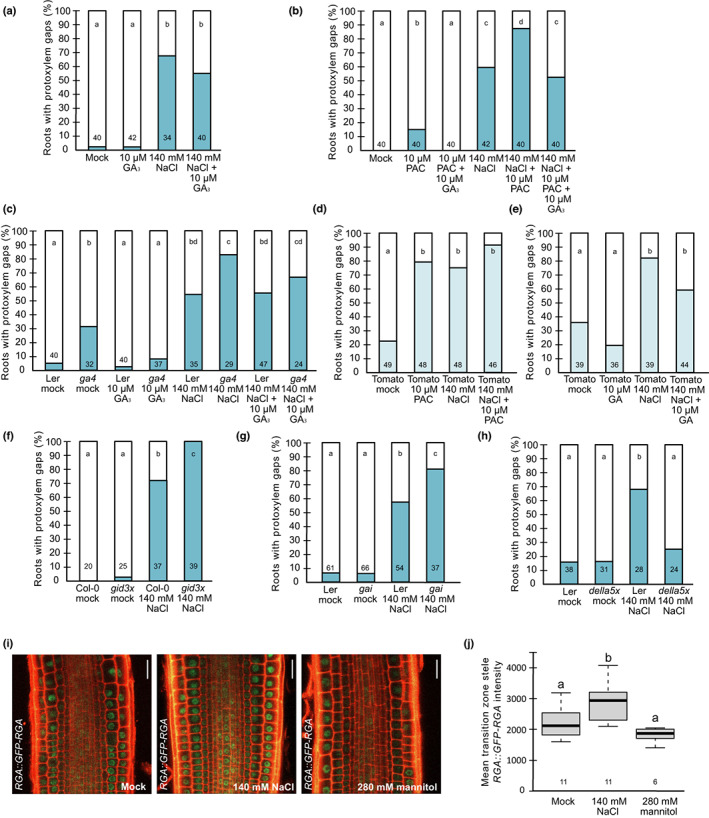
Reduced gibberellin (GA) levels and signalling induce protoxylem gap formation. (a–h) Quantification of roots exhibiting protoxylem gaps of 6‐d‐old Arabidopsis or *c*. 6‐d‐old tomato seedlings of the indicated genotypes grown under indicated conditions for 3 d. *Gid3x* is *gid1a‐2 gid1b‐3 gid1c‐1*; *della5x* is *gai‐t6 rga‐t2 rgl1‐1 rgl2‐1 rgl3‐4*. Numbers in bars are *n*; letters show statistical significance with multiple Fisher's exact test and Benjamini–Hochberg (BH) correction, *P* < 0.05. (i) Confocal micrographs of *pRGA::GFP‐RGA* in root transition zone of 5‐d‐old Arabidopsis seedlings after 6–9 h on 140 mM NaCl, 280 mM mannitol or mock conditions. Red, propidium iodide (PI) stain; Green, GFP. Bar = 20 μm. (j) Quantification of mean stele *pRGA::GFP‐RGA* intensity. Horizontal lines indicate median, whiskers indicate 1.5 interquartile range (IQR), numbers indicate *n*; letters indicate statistical significance with two‐way ANOVA, *P* < 0.05.

Several studies have indicated that levels of bioactive GAs are reduced under saline conditions (Magome *et al*., 2008; Colebrook *et al*., 2014). We therefore tested if altered GA levels could affect protoxylem gap formation upon growth on salt in Arabidopsis. For this, we first grew plants on either 10 μM GA_3_, 1 μM GA_4+7_ or on paclobutrazol (PAC), which inhibits GA biosynthesis (Lee *et al*., 1985), with and without 140 mM NaCl. Growth on GA alone did not affect xylem development but, when we combined GA and salt, we repeatedly noted a tendency of a lower number of plants forming protoxylem gaps, compared with salt‐grown plants without GA added (Figs 3a, S4a,b). PAC on its own, and in particular PAC together with salt, significantly enhanced the number of plants forming xylem gaps (Figs 3b, S4c). These effects were not observed when PAC was added together with GA_3_, confirming that the effect of PAC on protoxylem gap formation both under control conditions and on salt was due to its effect on GA levels (Figs 3b, S4c). Consistent with these findings, the *ga4* mutant (Koornneef & van der Veen, 1980; Talon *et al*., 1990), defective in a late step in the synthesis of bioactive GA, displayed protoxylem gaps that could be restored by the addition of GA_3_ (Figs 3c, S4d). Upon growth on salt, a significantly higher frequency of the mutant plants formed protoxylem gaps compared with wild‐type (Figs 3c, S4d). Similarly, *ga1‐3* and *ga1‐5*, defective in an earlier GA‐biosynthesis step (Sun *et al*., 1992), displayed an increased frequency of protoxylem gap formation upon growth on salt, although not when grown under control conditions (Figs S4e,f). Therefore, these findings suggested that the reduction in GA levels was linked to the protoxylem gap phenotype in Arabidopsis.

To test if gap formation occurred via similar mechanisms as in Arabidopsis, we treated tomato seedlings with 10 μM PAC or 10 μM GA_3._ As in Arabidopsis, the PAC treatment induced protoxylem gap formation in more plants than in the control, whereas GA_3_ had a lesser effect, with a tendency to reduce the number of plants forming protoxylem gaps both under control conditions and upon treatment with 140 mM NaCl (Fig. 3d,e). These results suggested that GA levels played a role in protoxylem gap formation in tomato similarly to that in Arabidopsis.

### DELLAs are required for protoxylem gap formation upon salt

Gibberellin is sensed by GID receptors and, consistent with our findings on altered GA levels, the *gid1a‐2 gid1b‐3 gid1c‐1* triple mutant, defective in GA perception (Griffiths *et al*., 2006), displayed both increased frequency of plants with protoxylem gaps and of gaps per root when grown on salt (Figs 3f, S5a). Upon GA perception, DELLAs, which act as transcriptional co‐regulators, become degraded to allow GA responses (Locascio *et al*., 2013). The DELLA‐protein GAI is stabilised in the *gai* mutant and therefore acts as a constitutive GA‐signalling repressor (Koorneef *et al*., 1985). Consistent with the involvement of DELLA‐mediated signalling for xylem gap formation, this mutant displayed an enhanced frequency of xylem gap‐forming plants upon salt (Fig. 3g). Previously, it was shown that the two DELLA proteins, RGL3 and RGA, are stabilised upon exposure to salt (Achard *et al*., 2006; Geng *et al*., 2013; Shi *et al*., 2017). To analyse if growth on salt may affect RGA accumulation also in the stele of the transition zone and the root meristem, we grew the *pRGA::GFP‐RGA* translational reporter lines on 140 mM NaCl or 280 mM mannitol and analysed GFP signal intensity. This revealed that salt, but not mannitol, significantly enhanced RGA accumulation in the vascular stele cells of the transition zone and the root meristem (Figs 3i,j, S5f,g), consistent with the previous study.

The five DELLAs appeared to function redundantly in the control of xylem gap formation. The single *della* mutants *gai‐td1* (Sessions *et al*., 2002), *rgl3‐5* and *rga‐28* (Tyler *et al*., 2004) had no effect on gap formation upon salt stress (Fig. S5b,c), whereas higher order mutants such as *rgl3‐5 rga‐28* and the *gai‐t6 rga‐t2 rgl1‐1 rgl2‐1* (*della4x*) mutant (Cheng *et al*., 2004), led to a gradual increase in the suppression of protoxylem gap formation (Fig. S5d,e). The DELLA quintuple (*della5x*) *gai‐t6 rga‐t2 rgl1‐1 rgl2‐1 rgl3‐4* mutant, defective in all five DELLA genes (Koini *et al*., 2009), did not form significantly more protoxylem gaps upon salt stress than under mock conditions (Fig. 3h). This suggests that protoxylem gap formation induced by high salinity requires multiple DELLA proteins.

### DELLA‐activated VND6 contributes to protoxylem gap formation upon salt stress

**Fig. 4:**
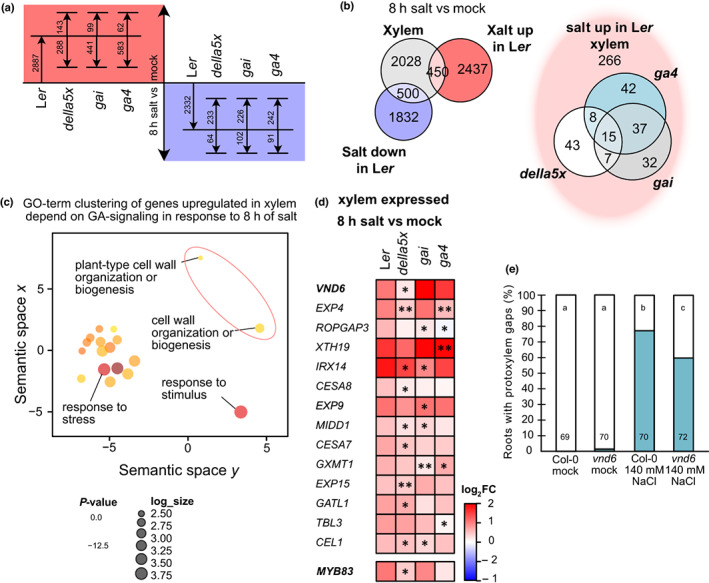
Cell wall‐related genes are differentially expressed in a DELLA‐dependent manner upon salt. (a) Differentially expressed genes (log_2_ FC < −0.5/> 0.5, *P*_adj_ < 0.05) after 8 h of salt exposure in L*er* (wildtype) roots and if indicated genotypes are significantly higher or lower expressed than L*er* (*P* < 0.05). *Della5x* is *gai‐t6 rga‐t2 rgl1‐1 rgl2‐1 rgl3‐4*. (b) Left: Venn diagram of genes differentially expressed, up or down, in L*er* after 8 h of salt exposure and genes expressed in xylem according to published single cell datasets (Denyer *et al*., 2019; Wendrich *et al*., 2020). Right: Venn diagram of xylem expressed genes upregulated by salt and differentially expressed (up or down) in *della5x*, *ga4* and/or *gai*. (c) Revigo clustering (Supek *et al*., 2011) of GO terms enriched among genes upregulated by 8 h salt exposure, xylem expressed and differentially expressed in *della5x*, *gai* and/or *ga4*. (d). Heatmap of genes with differential expression relative to L*er* within the GO term clusters related to cell wall organization or biogenesis from (c), as well as *MYB83* which is in the dataset, but not highlighted in the GOs. Bold font indicates master regulators of xylem development; *, *P* < 0.05; **, *P*_adj_ < 0.05. Statistical significance from DESeq2 analysis including a combinatorial effect (~genotype + genotype : condition). (e) Quantification of Col‐0 (wildtype) and *vnd6* roots exhibiting protoxylem gaps. Numbers indicate *n*; letters statistical significance with multiple Fisher's exact test and Benjamini–Hochberg (BH) correction, *P* < 0.05.

To further understand the processes regulated by altered GA levels and DELLA proteins in roots under salt stress, we analysed global gene expression changes in roots of 5‐d‐old wild‐type, *della5x*, *gai* and *ga4* seedlings after exposure to 140 mM NaCl for 1 h and/or 8 h compared with control conditions (Figs 4a, S6d). The time points were selected to show the response at the initial stress response phase, and when the plants had acclimatised and resumed growth and development (Geng *et al*., 2013). Corroborating our results suggesting that salt reduced levels of active GAs in the roots, we found several GA‐2 oxidases, which inactivate bioactive GAs, upregulated upon exposure to salt in the initial stress response phase (Table S2). As salt affects protoxylem differentiation, we focused our attention on genes previously found to be expressed in immature xylem in two different single cell transcriptomes (Denyer *et al*., 2019; Wendrich *et al*., 2020). Consistent with the notion that a subset of the early stress responding genes are under DELLA regulation, we found GO terms such as ‘response to stress’, ‘response to stimulus’ and ‘response to other cellular components’ enriched among genes that were upregulated in wild‐type and differentially expressed in the *della5x* mutant, among the xylem‐active genes (Table S3). Genes downregulated in wild‐type upon salt exposure, differentially regulated by *della5x* and expressed in immature xylem were enriched for processes such as ‘genes encoding enzymes involved in hemicellulose synthesis’ (Fig. S6e–g). Therefore, cell wall modifications may happen rapidly, but we expected most of the factors underlying gap formation to primarily express at the acclimation phase at 8 h. At the 8 h time point, 2887 genes were activated after salt exposure in wild‐type (log_2_FC > 0.5/log_2_ FC < −0.5, *P*_adj_ < 0.05). Of these 450 had a reported xylem expression, of which 184 were differentially expressed in *della5x*, *ga4* and/or *gai* (*P* < 0.05) (Fig. 4b). These genes were enriched in GO terms related to ‘response to stress’ but also ‘cell wall organisation and biosynthesis’ (Fig. 4c), whereas the corresponding downregulated genes included, for example, ‘stress response’, ‘water transport’ and ‘cellular processes’ (Fig. S6a,b). As the cell walls were altered in the protoxylem gap cells, we focused our attention on the ‘cell wall organisation and biosynthesis’ group of genes (Fig. 4d). Among these were several encoding proteins with functions related to cellulose and hemicellulose biosynthesis, along with the xylem master regulator VND6, as well as several alpha‐expansins. The xylem expressed list with differential expression in GA mutants included also the previously described VND6 target *MYB DOMAIN PROTEIN 83* (*MYB83*) (McCarthy *et al*., 2009; Fig. 4d). Whereas these genes were activated by salt, the *della5x* mutant displayed less strong activation and/or expression was further upregulated in *gai* or *ga4*. This result is in agreement with our finding that *della5x* suppressed xylem gap formation, and also indicated that one or more of the DELLAs directly or indirectly activates transcription of these xylem regulator genes in the wild‐type under salt stress. Several genes that were activated under salt stress, but less so in the *della5x* mutant, including *VND6*, displayed instead elevated expression levels in *della5x* compared with wild‐type under control conditions (Fig. S6c; Table S2).

VND6 is reported to be active in metaxylem and as a metaxylem master regulator (Kubo *et al*., 2005). However, recent single cell analyses highlights also its activity in protoxylem cells (Fig. S6h) To examine the potential influence of VND6 on xylem development upon salt stress, we analysed the *vnd6* mutant (Kubo *et al*., 2005). Although it did not exhibit any apparent differences in xylem development under control conditions, significantly less protoxylem gaps were formed in *vnd6* upon salt stress compared with wild‐type (Fig. 4e), suggesting a role for VND6 in salt‐induced protoxylem gap formation. The gap reduction in *vnd6* was relatively limited, indicating that additional factors contributed to the xylem gap formation under salt. VND7 is a VND6 paralogue, a well known regulator of protoxylem development (Kubo *et al*., 2005), and therefore another candidate for the observed protoxylem phenotype. However, *VND7* expression was not changed in our analyses and, in contrast with *vnd6*, *the vnd7* mutant did not affect protoxylem gap formation upon salt stress (Fig. S6i).

### Expansins may be involved in DELLA‐dependent xylem gap formation

**Fig. 5:**
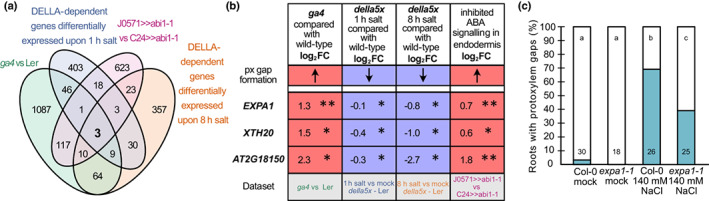
Cell wall modifying enzymes in xylem gap formation. (a) Venn diagram showing the overlap between different xylem gap‐related datasets: *ga4* vs L*er* (this study, log_2_FC < −0.5/> 0.5, *P* < 0.05), DELLA‐dependent genes differentially expressed upon 1 h salt (this study; L*er* log_2_FC > 0.5/− < 0.5, *P*_adj_ < 0.05; *della5x* (*gai‐t6 rga‐t2 rgl1‐1 rgl2‐1 rgl3‐4*) *P* < 0.05), DELLA‐dependent genes differentially expressed upon 8 h salt (this study; L*er* log_2_FC > 0.5/− < 0.5, *P*_adj_ < 0.05; *della5x P* < 0.05), and *J0571>>abi1‐1* vs *C24>>abi1‐1* (Ramachandran *et al*., 2021; log_2_FC > 0.5/−< 0.5, *P* < 0.05). (b) Differential expression relative to wild‐type (log_2_FC) of genes shared by the four datasets, shown in (a). *, *P* < 0.05; **, *P*_adj_ < 0.05. Statistical significance from DESeq2 analysis including a combinatorial effect (~genotype + genotype : condition). (c) Quantification of roots exhibiting protoxylem gaps of 6‐d‐old Arabidopsis seedlings of Col‐0 (wild‐type) and *expa1‐1* upon growth on 140 mM NaCl or under mock conditions for 3 d. Numbers indicate *n*; letters indicate statistical significance with multiple Fisher's exact test and Benjamini–Hochberg (BH) correction, *P* < 0.05.

Xylem gaps were not only observed upon salt stress, but certain genotypes displayed gaps also under normal conditions. This included protoxylem gaps in *ga4* (Fig. 3c) and metaxylem gaps upon inhibition of ABA signalling in the ground tissue caused by driving *abi1‐1* by the ground tissue‐specific J0571 enhancer line (*J0571>>abi1‐1* vs *C24>>abi1‐1*; Ramachandran *et al*., 2021). We searched for a common set of genes that were differentially expressing in genotypes exhibiting xylem gaps and in our salt‐treatment datasets, and identified three genes: *EXP1*, *XYLOGLUCAN ENDOTRANSGLUCOSYLASE/HYDROLASE* (*XTH20*) and a peroxidase family gene (*At2G18150*) (Fig. 5a,b). Neither *EXP1* nor *XTH20* were found in the immature xylem single cell transcriptomes (Denyer *et al*., 2019; Wendrich *et al*., 2020), but previous expression data indicated that both were upregulated in the stele in response to salt (Fig. S7a) (Geng *et al*., 2013). As several genes encoding expansins were upregulated in response to salt in our analyses (Figs 4d, S7b) and expansins previously had been related to salt stress tolerance and vascular development (Jadamba *et al*., 2020), we focused on EXP1. Whereas the *expa1‐1* mutant did not display any xylem deviations under normal conditions, this mutant could partially suppress protoxylem gap formation upon salt stress (Fig. 5c), linking EXP1 also to modifications in xylem development upon salt stress. To test the link between GA and *VND6* and *EXP1*, we treated the mutants with PAC. Both *vnd6* and *expa1‐1* mutants displayed subtle tendencies to reduce the effect of PAC (Fig. S7c), in line with VND6 and EXP1 acting downstream of GA signalling.

Taken together, these results connected the xylem developmental regulator VND6, along with several of its well known targets including genes encoding SCW‐modifying enzymes, as well as with one or more alpha‐expansins as GA‐regulated factors important for the formation of protoxylem gaps under salt stress. The relatively subtle effect of *vnd6* on gap formation in response to salt and PAC further indicates that other, as of yet unidentified, transcriptional regulators also are important for intermittent repression of xylem differentiation.

### Xylem gaps correlate with better survival under salt stress

**Fig. 6:**
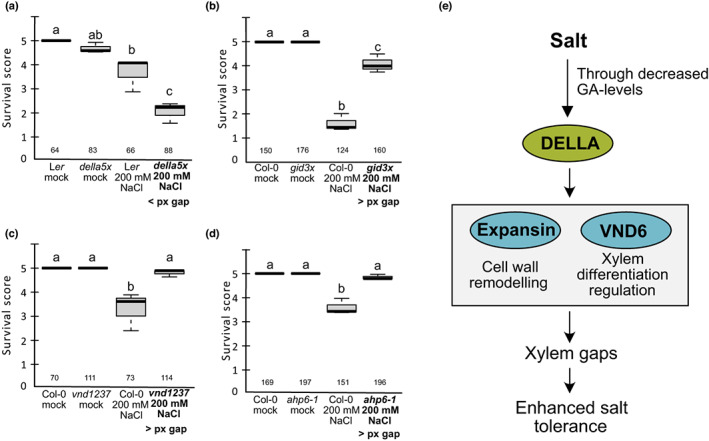
Enhanced protoxylem gap formation confers increased salt tolerance. (a–d) Survival after growth on 200 mM NaCl or under mock conditions for 4 d for mutants with more (*gid3x*, *gid1a‐2 gid1b‐3 gid1c‐1*; *vnd1237, vnd1 vnd2 vnd3 vnd7,* and *ahp6‐1*) or less (*della5x*, *gai‐t6 rga‐t2 rgl1‐1 rgl2‐1 rgl3‐4*) protoxylem (px) gap formation in response to salt stress, compared with wild‐type (Col‐0 or L*er*). Survival score was calculated by assigning plants with white cotyledons a score of 1, pale green 3 and green 5. These scores were multiplied and then divided by *n* analysed plants. Horizontal lines indicate median, whiskers indicate 1.5 interquartile range (IQR), numbers indicate *n* from three replicates. Letters indicate statistical significance with two‐way ANOVA, *P* < 0.05. (e) Proposed model for the molecular regulation of xylem gap formation in response to salt stress. Salt causes a decrease in levels of bioactive gibberellins (GAs) (Achard *et al*., 2006) leading to the stabilisation of DELLA proteins. One or more of the DELLAs promote, among others, expression of the xylem development master regulator *VND6*, along with genes for SCW formation, expansins and other cell wall remodelling genes, resulting in the occasional prevention of fully differentiated protoxylem cells, visible as protoxylem gaps, by a yet unknown mechanism. These gaps promote enhanced salt tolerance in young seedlings.

Next, we asked if the xylem gaps might help the plant withstand salt stress. As we had observed enhanced and reduced protoxylem gap formation in the *gid3x* and the *della5x* mutants, respectively (Fig. 3f,h), we first tested the salt tolerance of these, along with the *expa1* and *vnd6* mutants, displaying partially suppressed gap formation (Figs 4e, 5c). We grew 3‐d‐old seedling mutants along with wild‐type on medium containing 200 mM NaCl for 4 d, and then scored the survival of the 7‐d‐old plants by the colour of the cotyledons (white vs green or pale green). The survival rate of the *della5x* mutant was significantly worse than wild‐type upon salt stress, whereas *gid3x* survived significantly better upon salt stress compared with wild‐type (Figs 6a,b, S8a,b). For the *vnd6* and *expa1‐1* mutants we did not observe a significant difference in tolerance compared with wild‐type (Fig. S8h–j). We noted that treatment with 200 mM NaCl severely compromised root growth (Fig S8a,b,c,f,i). Therefore, the formation of new xylem is limited under these extreme conditions. Furthermore, it has previously been reported that DELLAs promote salt tolerance by reducing reactive oxygen species (ROS) levels (Achard *et al*., 2008). Therefore, it is possible that the effects on survival observed in the GA‐signalling mutants were independent of their capacity for xylem gap formation.

To further test the hypothesis that xylem gaps promote salt tolerance, we next assessed the salt tolerance of genotypes with no known connection to GA signalling, but exhibiting excessive protoxylem gap formation under normal conditions. We selected *ahp6‐1*, which affects protoxylem specification by interfering with the auxin–cytokinin balance (Mähönen *et al*., 2006) and a multiple *vnd* mutant, *vnd1 vnd 2 vnd3 vnd7* (*vnd1237*), displaying both protoxylem and metaxylem gaps, along with the *vnd7* mutant showing no deviant xylem phenotypes under normal conditions (Tan *et al*., 2018; Ramachandran *et al*., 2021; Figs S6i, S8d,e,g). Both *ahp6‐1* and *vnd1237* survived significantly better upon salt stress compared with the wild‐type, whereas *vnd7* behaved like the wild‐type (Figs 6c,d, S8c,f,j). We next tested if also older plants benefitted from extensive protoxylem gaps, and analysed tolerance of *ahp6‐1* transferred to salt when 7‐d old, and analysed the survival in 11‐d‐old plants, exposed to high salt for 4 d. At this age we did not find a significant difference in salt stress tolerance compared with the wild‐type (Fig. S8l), indicating that protoxylem gaps may be particularly important for young seedlings.

We next attempted to test if we could associate gap formation in response to salt with tolerance. Because of the severe effects of 200 mM NaCl we instead assessed the effects of prolonged exposure to 140 mM NaCl, which allows root growth, in Col and L*er* seedlings. Interestingly, for both wild‐type ecotypes there was a significantly higher amount of plants displaying protoxylem gaps and green cotyledons compared with those with white cotyledons (Fig. S8k), showing a correlation with xylem gap formation in response to salt and salt tolerance.

## Discussion

### Protoxylem gaps may promote seedling survival under high salinity

High soil salinity strongly impairs crop productivity and is a major problem in agriculture (Shannon & Grieve, 1999). Although salt affects plants at all developmental stages, the seedling establishment phase may be particularly vulnerable, and genetic traits affecting the performance of early life stages contribute strongly to selection and local adaptation (Postma & Ågren, 2016). Here, we provide results showing that the extent to which young seedlings form protoxylem gaps correlates with salt tolerance. We cannot exclude that other parameters also influence salt tolerance but, supporting the relevance of protoxylem gaps, such gaps are induced by high salinity not only in wild‐type Arabidopsis seedlings (Col‐0 and L*er* ecotypes), but also in seedlings of other eudicot species. Previous studies have found that processes that inhibit the transport of salt ions to the shoot via the xylem promote salt tolerance (Møller *et al*., 2009; Jiang *et al*., 2012). This may be seen in relatively salt tolerant Arabidopsis accessions that respond to salt by developing smaller vessels and less well developed xylem compared with phloem in the stem, potentially reducing hydraulic conductivity (Sellami *et al*., 2019a). The formation of protoxylem gaps may be another measure to prevent or delay salt ion distribution within the seedling. By contrast, the *acl5* mutant that developed extensive xylem was salt hypersensitive (Shinohara *et al*., 2019). The protoxylem gaps formed in response to salt stress may be particularly important during the seedling establishment phase, as 11‐d‐old *ahp6* seedlings with excessive protoxylem gaps, but after the onset of secondary growth (Smetana *et al*., 2019), behaved similarly to wild‐type plants on salt. In very young seedlings, water transport relies relatively more on the protoxylem, whereas most transport would occur via metaxylem and secondary xylem vessels in later developmental stages, probably decreasing the relative relevance of the protoxylem strands.

### Protoxylem gap formation upon high salinity requires reduced GA signalling

Previously, we and others have found that plants exposed to water‐limiting conditions or elevated ABA levels respond by forming additional protoxylem strands and metaxylem closer to the root tip (Ramachandran *et al*., 2018, 2021; Bloch *et al*., 2019). Here we show that these ABA‐mediated responses also occur upon exposure to salt stress, but only relatively late. A more rapid response is the formation of protoxylem gaps, which happens independently of ABA signalling. Instead, multiple lines of evidence point towards reduced GA levels and/or signalling as critical for the formation of protoxylem gaps in response to salt stress. In particular, a mutant defective in all five DELLA genes, and therefore with de‐repressed GA signalling, did not form protoxylem gaps upon salt stress. Previous data have shown that high levels of GA render plants more sensitive to salt, and that GA‐2‐oxidases, which normally are upregulated by salt, reduce GA levels and protect against salt stress (Achard *et al*., 2006; Magome *et al*., 2008; Colebrook *et al*., 2014). Consequently, *della* mutants are less salt tolerant, whereas stabilisation of RGL3, along with the auxin‐signalling repressor IAA17, confers salt stress resistance (Achard *et al*., 2006, 2008; Shi *et al*., 2017). Whereas multiple studies connect GA signalling with various steps of xylem development (Ashraf *et al*., 2002; Colebrook *et al*., 2014; Guo *et al*., 2015; Yamazaki *et al*., 2018; Singh *et al*., 2019), it has not been clear if GA's effects on xylem morphology and differentiation impact salt tolerance. Therefore, our findings here contribute a link between GA's effects on xylem differentiation and salt sensitivity.

### Xylem master regulator VND6 is used in gap formation under salt stress

A relatively large set of previously identified xylem‐active genes were upregulated in a DELLA‐dependent manner under salt, including genes encoding the xylem differentiation master regulators VND6 and MYB83 (Kubo *et al*., 2005; McCarthy *et al*., 2009), along with multiple genes known to act downstream of these regulators encoding, for example, SCW cellulose synthases. This was a puzzling observation, as the protoxylem gaps had reduced SCW differentiation. However, the *vnd6* mutant displayed a reduced capacity for gap formation, suggesting that the activation of *VND6* upon salt indeed was connected to intermittently inhibited protoxylem cell differentiation. Therefore, these results suggested that *VND6* activated under salt conditions may have a different and additional role rather than governing metaxylem differentiation, and instead contribute to the modification of protoxylem differentiation. How VND6 orchestrates this feat is currently unknown, and will be an important question for future research. Intriguingly, our transcriptome data suggest that DELLAs repressed *VND6* under control conditions, but activated it upon salt stress. It is known that DELLAs can act both as transcriptional activators and repressors depending on the interaction partner (Locascio *et al*., 2013; Yoshida *et al*., 2014). Discontinuous xylem has previously been observed when overexpressing WRKY15 (Ge *et al*., 2020). In our transcriptome, we see an induction of *WRKY15* upon growth on salt, however this appears to be DELLA independent. It will be relevant to further assess a potential connection between WRKY15 and VND6, as well as identifying DELLA interacting partners under normal and high salt conditions to elucidate how VND6 may shift activity to contribute to the promotion of protoxylem gaps under salt stress.

It is likely that other factors in addition to VND6 act downstream of the DELLAs, as the *vnd6* mutant could not completely suppress gap formation under salt. We found that a mutant of *EXP1* also partially could repress xylem gap formation, indicating that cell wall remodelling also contributes to xylem gap formation. Previous studies have similarly found elevated expansin levels under salt stress, and cell walls may undergo extensive remodelling under salt acclimation (Shen *et al*., 2014). Altered SCW composition with a higher cellulose and hemicellulose content and reduced lignin was observed in the Arabidopsis stem after salt stress, resulting in enhanced vessel elasticity, and preventing collapse due to the osmotic stress (Sellami *et al*., 2019b). In rice, overexpression of *OsEXPA7* led to changes in the vasculature and increased salt tolerance (Jadamba *et al*., 2020). From our results, we cannot tell if cell wall modifications happened exclusively in the xylem or also in other tissues, and it will be relevant to further assess cell‐specific effects upon salt stress.

### Conclusion

Salt stress induces the local inhibition of protoxylem differentiation, causing protoxylem gaps in young Arabidopsis seedlings. Gap formation requires the DELLA‐mediated repression of GA signalling, and it is likely that salt stress triggers a reduction in bioactive GA levels, therefore stabilising DELLA proteins. Under salt stress, DELLAs promote a set of VND6‐regulated factors, which may include SCW differentiation enzymes. DELLAs also promote other enzymes, such as alpha‐expansins, as depicted in the model in Fig. 6d. Mutational analysis of *VND6* and *EXP1* linked these factors to the formation of protoxylem gaps under salt, as the mutants partially suppressed gap formation. The xylem gaps may confer salt tolerance to young seedlings and therefore provide an adaptive advantage. Seedlings of several other, distantly related, eudicot species similarly formed protoxylem gaps upon salt stress, and tomato seedlings responded similarly to Arabidopsis to reduced GA levels. These findings suggested that protoxylem gap formation and the mechanisms governing this trait are not specific to Arabidopsis.

## Competing interests

None declared.

## Author contributions

Design of the research, FA, AC; performance of the research, FA; data analysis, FA, AC; writing – original draft, FA; writing – review and editing, FA, AC; funding acquisition, FA, AC.

## Supporting information


**Fig. S1** Protoxylem gaps are formed in response to salt.
**Fig. S2** Protoxylem gap formation is not abscisic acid mediated.
**Fig. S3** Protoxylem gaps are formed in several eudicot species upon salt.
**Fig. S4** Reduced gibberellin levels induce protoxylem gap formation.
**Fig. S5** Reduced gibberellin signalling induces protoxylem gap formation.
**Fig. S6** Cell wall‐related genes are differentially expressed in a DELLA‐dependent manner upon salt.
**Fig. S7** Cell wall modifying enzymes in xylem gap formation.
**Fig. S8** Enhanced protoxylem gap formation confers increased salt tolerance.
**Methods S1** Key resources used in this study.

**Table S1** Summary of statistical analyses.

**Table S2** Differentially expressed genes in roots of L*er*, *della5x*, *gai* and *ga4* upon growth on salt for 1–8 h.
**Table S3** Enriched gene ontologies from Panther analysis of xylem expressed genes upregulated and downregulated by 1 h/8 h of salt exposure in DELLA‐/gibberellin‐dependent manner.
Please note: Wiley Blackwell are not responsible for the content or functionality of any Supporting Information supplied by the authors. Any queries (other than missing material) should be directed to the *New Phytologist* Central Office.

## Data Availability

All data and material produced in this study are available on request from the corresponding author.
