# Supplementary material for: Salinity induces discontinuous protoxylem via a DELLA‐dependent mechanism promoting salt tolerance in Arabidopsis seedlings
Source: New Phytol. 2022 Jul 12;236(1):195–209. doi: 10.1111/nph.18339 (PMC9545557; doi:10.1111/nph.18339)
Supplement: Supplementary file 1 — Fig. S1 Protoxylem gaps are formed in response to salt. Fig. S2 Protoxylem gap formation is not abscisic acid mediated. Fig. S3 Protoxylem gaps are formed in several eudicot species upon salt. Fig. S4 Reduced gibberellin levels induce protoxylem gap formation. Fig. S5 Reduced gibberellin signalling induces protoxylem gap formation. Fig. S6 Cell wall‐related genes are differentially expressed in a DELLA‐dependent manner upon salt. Fig. S7 Cell wall modifying enzymes in xylem gap formation. Fig. S8 Enhanced protoxylem gap formation confers increased salt tolerance. Methods S1 Key resources used in this study. [file NPH-236-195-s002.pdf]

New Phytologist

***New Phytologist* Supporting Information**

Article title: **Salinity induces discontinuous protoxylem via a DELLA-dependent mechanism promoting salt tolerance in Arabidopsis seedlings**

Authors: Frauke Augstein and Annelie Carlsbecker

Article acceptance date: 11 June 2022

The following Supporting Information is available for this article:

Fig. S1 Protoxylem gaps are formed in response to salt.

Fig. S2 Protoxylem gap formation is not ABA mediated.

Fig. S3 Protoxylem gaps are formed in several eudicot species upon salt.

Fig. S4 Reduced GA-levels induces protoxylem gap formation.

Fig. S5 Reduced GA-signalling induces protoxylem gap formation.

Fig. S6 Cell wall related genes are differentially expressed in a DELLA-dependent manner upon salt.

Fig. S7 Cell wall modifying enzymes in xylem gap formation.

Fig. S8 Enhanced protoxylem gap formation confers increased salt tolerance.

Table S1 Summary of statistical analyses (see separate file)

Table S2 Differentially expressed genes in roots of L*er*, *della5x*, *gai* and *ga4* upon growth on salt for 1h and 8h, related to Fig. 4, 5, S6, S7. (see separate file)

Table S3 Enriched GOs from Panther analysis of xylem expressed genes up- and downregulated by 1h/ 8h of salt exposure in DELLA-/ GA-dependent manner

Methods S1 Key resources used in this study

Fig. S1 Protoxylem gaps are formed in response to salt. All data are from roots of 6-day old Arabidopsis seedlings grown for 3 days on NaCl, mannitol, ABA or under mock conditions, unless otherwise stated. **(a)** Cartoon of protoxylem gap formation along the root upon exposure to salt. **(b)** Root growth of Arabidopsis seedlings on NaCl or mock plates. Numbers above the graph indicate n roots analyzed; Horizontal lines indicate median, whiskers indicate 1.5 Interquartile range (IQR), letters indicate statistical significance with t-test, $p < 0.05$. **(c)** Quantification of number of primary root tips of 14 day old plants grown on 140mM NaCl for 3 days with protoxylem gaps. Number of analyzed roots (n) is indicated on the bars; letters indicate statistical significance with multiple Fisher's exact test and BH correction, $p < 0.05$ **(d)** Quantification of number of roots with protoxylem gaps after growth on different concentrations of salt or mannitol, where 280 mM mannitol is iso-osmolaric to 140 mM NaCl. **(e)** Seedlings were transferred for three days to Treatment 1 (T1) and then transferred to Treatment 2 (T2) for three days. The parts of the roots that were grown under the different treatments were collected and analyzed separately.

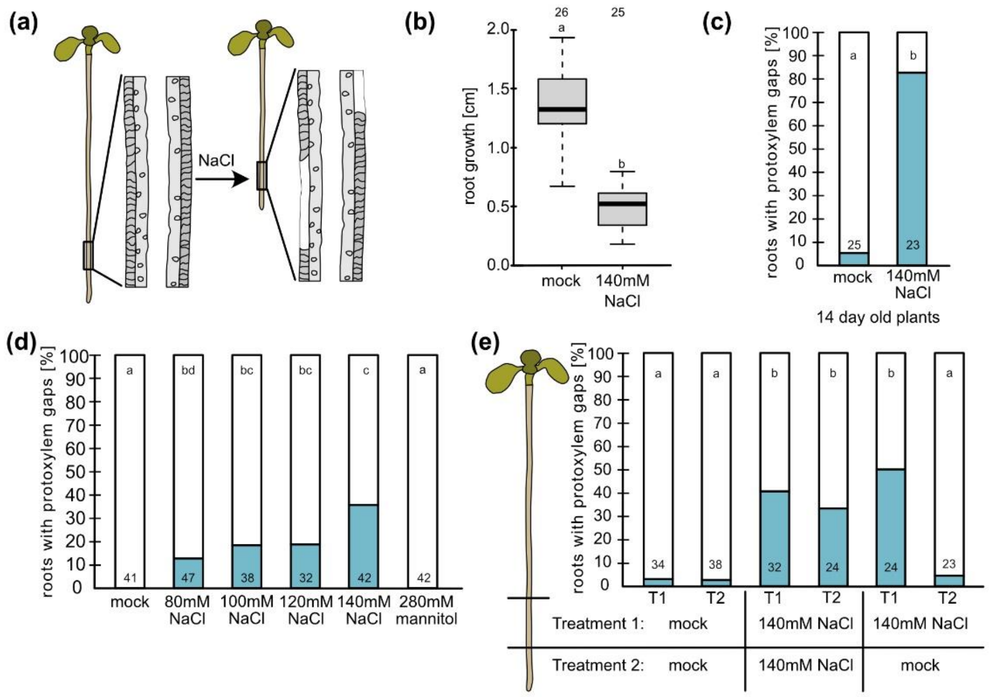

Fig. S2 Protoxylem gap formation is not ABA mediated. (a) Quantification of roots with protoxylem gaps after growth on salt for 3, 5 or 7 days. **(b)** Quantification of roots with extra protoxylem after growth on salt for 3, 5 or 7 days. **(c)** Quantification of extra protoxylem in wild type (Col-0) or *abi1-1C* roots after growth on salt or mannitol. **(d)** Differential interference (DIC) images of Col-0 root xylem. Scale bars, 50 µm **(e)** Quantification of roots exhibiting differentiated inner metaxylem (imx) at 1 mm from the root tip, after growth on salt or mannitol. **(f)** Root growth of Col-0 or *abi1-1C* on salt or mannitol. Horizontal lines indicate median, whiskers indicate 1.5 Interquartile range (IQR), letters indicate statistical difference with Two-way-ANOVA; $p < 0.05$. **(g)** Quantification of number of roots with protoxylem gaps in Col-0 and *abi1-1C* after growth on salt or mannitol. **(h)** Quantification of number of roots with protoxylem gaps in Col-0 after growth on mock or 1µM ABA for 3 days. **(i)** Quantification of roots with protoxylem gaps in Col-0 and *snrk2.2 snrk2.3* after growth on salt. **(j)** Root growth of Col-0 and *snrk2.2 snrk2.3* on salt. In **a**, **c-e**, **g-j,** horizontal lines indicate median, whiskers indicate 1.5 Interquartile range (IQR), numbers in bars indicate n; letters indicate statistical significance with multiple Fisher's exact test and BH correction, $p < 0.05$. In f and j, numbers in bars indicate n; letters indicate statistical significance with Two-way ANOVA, $p < 0.05$.

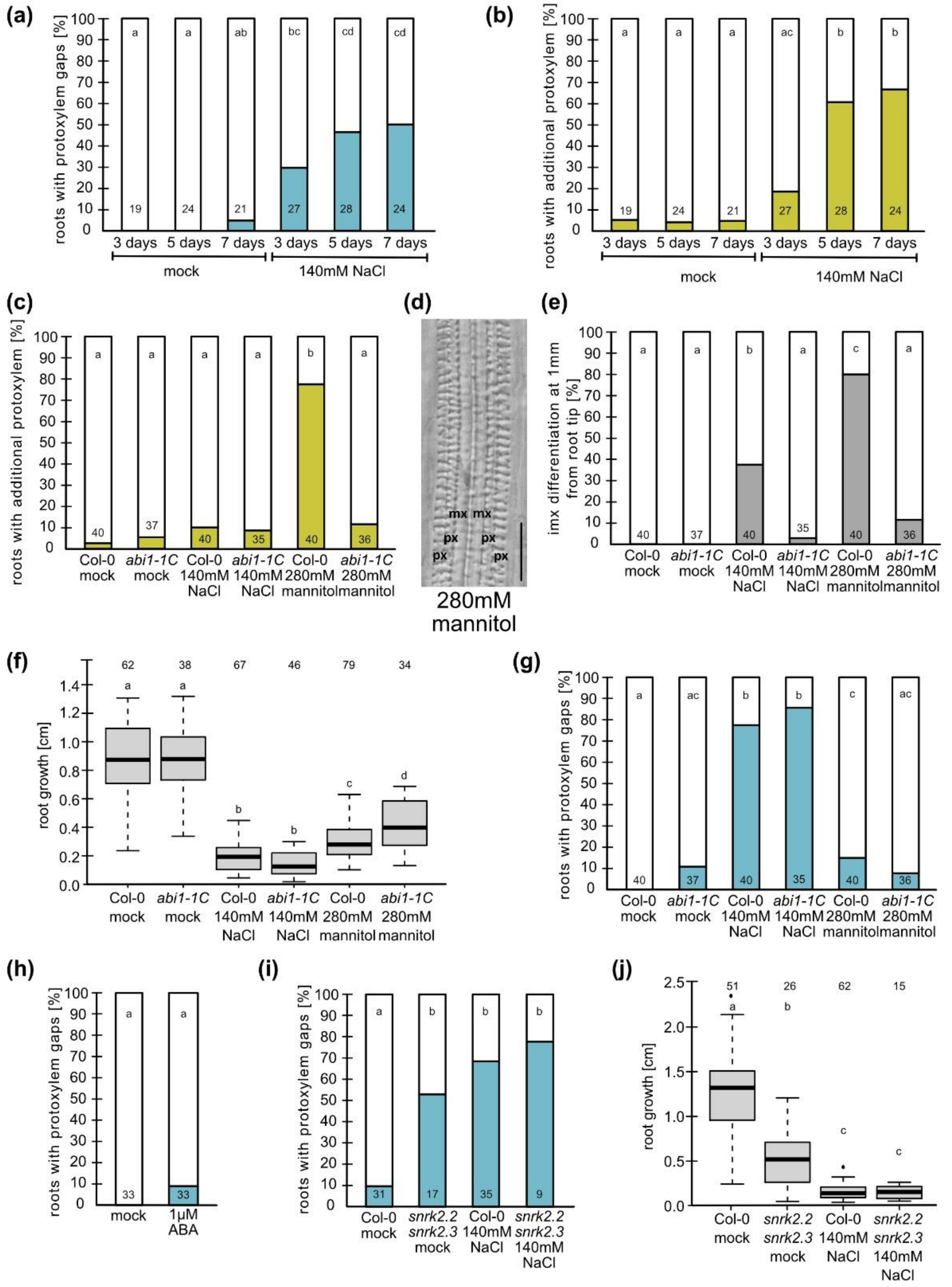

(a)
roots with protoxylem gaps [%]
3 days
5 days
7 days
mock
140mM NaCl
(b)
roots with additional protoxylem [%]
(c)
roots with additional protoxylem [%]
Col-0 mock
abi1-1C mock
Col-0 140mM NaCl
abi1-1C 140mM NaCl
Col-0 280mM mannitol
abi1-1C 280mM mannitol
(d)
mx
px
280mM mannitol
(e)
imx differentiation at 1mm from root tip [%]
(f)
root growth [cm]
(g)
roots with protoxylem gaps [%]
(h)
roots with protoxylem gaps [%]
mock
1µM ABA
(i)
roots with protoxylem gaps [%]
Col-0 mock
snrk2.2 snrk2.3 mock
Col-0 140mM NaCl
snrk2.2 snrk2.3 140mM NaCl
(j)
root growth [cm]

Fig. S3 Protoxylem gaps are formed in several eudicot species upon salt. (a) Quantification of sugar beet roots exhibiting protoxylem gaps after growth for 3 days on 140 mM NaCl. **(b)** Quantification of *Eutrema* roots exhibiting protoxylem gaps after growth for 3 days on 140 mM NaCl. **(c)** Quantification of *Eutrema* roots exhibiting extra protoxylem after growth for 3 days on 200 mM NaCl. Numbers in bars indicate n; letters indicate statistical significance with multiple Fisher's exact test and BH correction, $p < 0.05$.

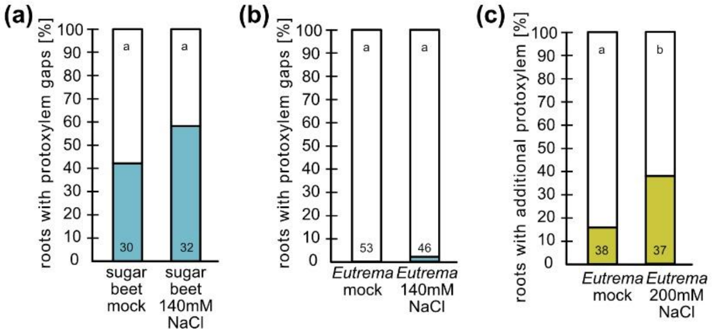

Fig. S4 Reduced GA-levels induces protoxylem gap formation. All results presented were observed in roots of 6-day old Arabidopsis seedlings of indicated genotypes grown for 3 days on 140mM NaCl or under mock conditions, and with indicated treatments of GA_3, GA_{4+7} (dissolved in DMSO) or Paclobutrazol (PAC). *Della4x* is *gai-t6 rga-t2 rgl1-1 rgl2-1*. **(a), (c - d)** Quantification of roots exhibiting different amount of protoxylem gaps. Numbers indicate n of the observed phenotype **(b)**, **(e - f)** Quantification of roots exhibiting protoxylem gaps. Numbers indicate n; letters indicate statistical significance with multiple Fisher's exact test and BH correction, $p < 0.05$.

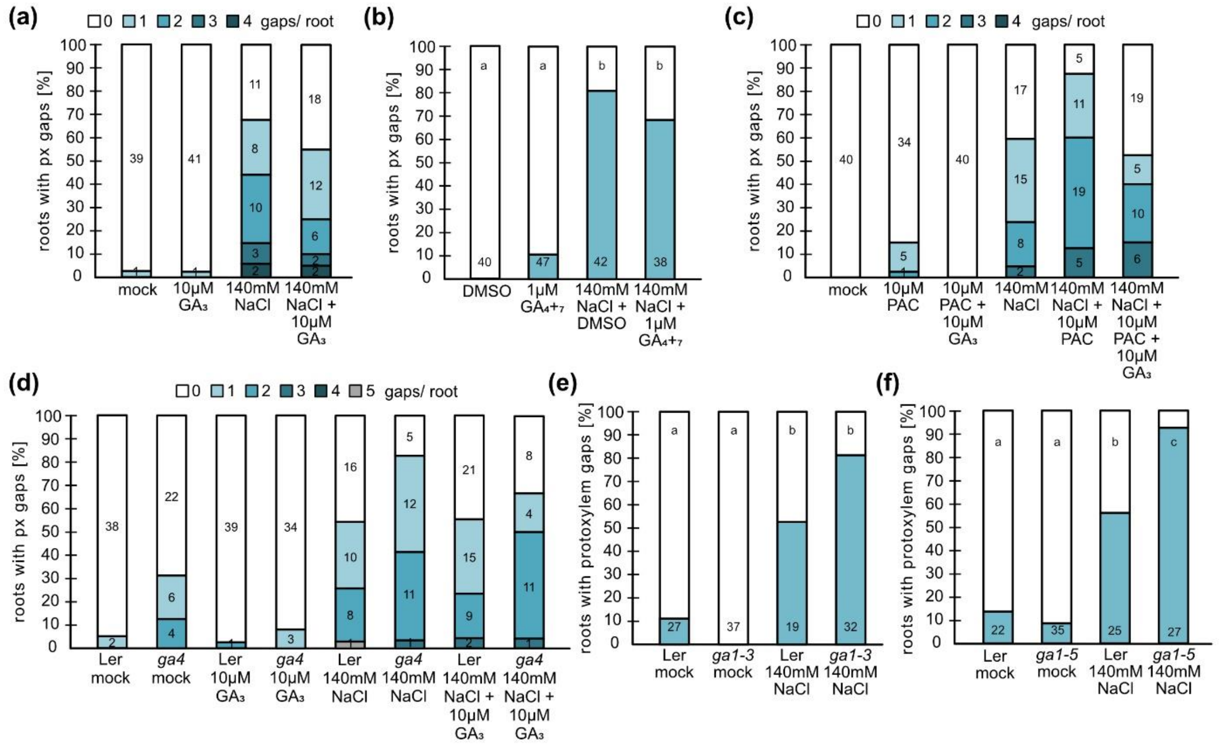

Fig. S5 Reduced GA-signalling induces protoxylem gap formation. (a) Quantification of roots exhibiting different amount of protoxylem gaps. Numbers indicate n of the observed phenotype **(b - e)** Quantification of roots exhibiting protoxylem gaps. Numbers indicate n; letters indicate statistical significance with multiple Fisher's exact test and BH correction, $p < 0.05$. **(f)** Confocal micrographs of *RGA::GFP-RGA* in root meristems of 5-day old Arabidopsis seedlings after 6-9 h on 140mM NaCl, 280mM mannitol or mock conditions. Purple, PI-stain; Green, GFP. Scale bar, 20µm. **(g)** Quantification of mean meristem stele *RGA::GFP-RGA* intensity. Horizontal lines indicate median, whiskers indicate 1.5 Interquartile range (IQR), dots above leftmost plot indicate outliers, numbers indicate n, letters statistical significance with Two-way ANOVA, $p < 0.05$.

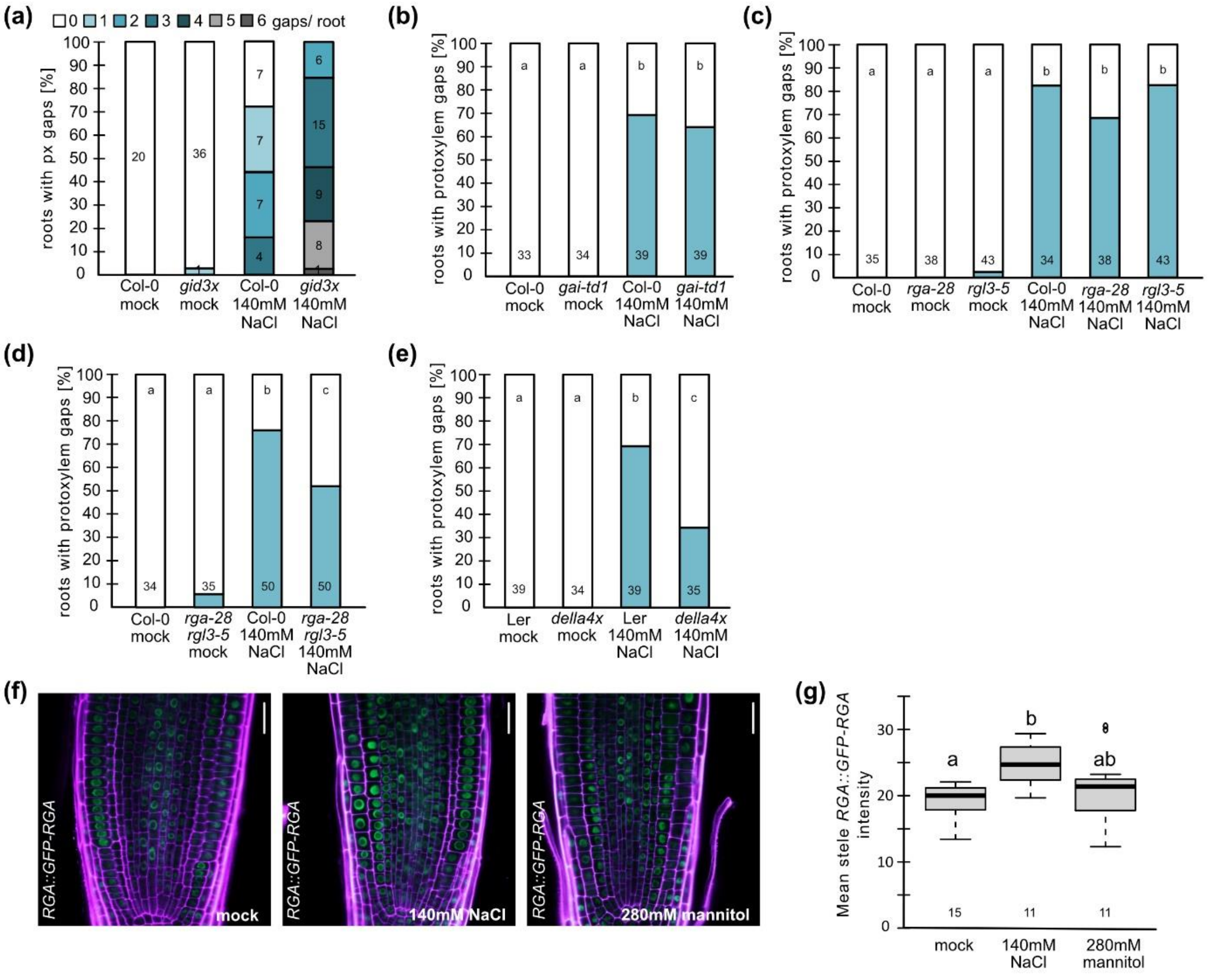

Fig. S6 Cell wall related genes are differentially expressed in a DELLA-dependent manner upon salt. (a) Venn diagram-section (light blue) of genes downregulated in wildtype and expressed in xylem according to published single cell data sets (Denyer et al., 2019; Wendrich et al., 2020), see fig. 4B. The fraction of genes differentially expressed in *della5x* (*gai-t6 rga-t2 rgl1-1 rgl2-1 rgl3-4*), *ga4* and/or *gai* is displayed on top of the section. **(b)** REVIGO clustering (Supek et al., 2011) of GO-terms enriched for genes that are downregulated upon salt, xylem expressed and differentially expressed in *della5x*, *gai* and/or *ga4*. **(c)** Heatmap of genes that are differentially regulated in della5x vs. wildtype under mock ($log2FC < -0.5$/ > 0.5, $p < 0.05$), and filtered for xylem-expression. **(d)** Differentially expressed genes ($log2FC < -0.5$/ >0.5, $padj < 0.05$) after 1h of salt exposure in Ler (wildtype) and the fraction of those genes differentially expressed in della5x ($p < 0.05$). **(e)** Venn diagram of genes that are up- and downregulated in wildtype after 1h of salt exposure and genes expressed in xylem according to published single cell data sets (Denyer et al., 2019; Wendrich et al., 2020). Venn diagram fractions display upregulated (pink) or downregulated (light blue) xylem expressed genes differentially expressed in della5x. **(f)** REVIGO clustering (Supek et al., 2011) of GO-terms enriched for genes that are upregulated upon 1h salt, xylem expressed and differentially expressed in della5x. **(g)** Heatmap of genes extracted from GO-term enrichment analysis for genes that are downregulated upon 1h salt. Only the three shown GO-terms were enriched. *, $p < 0.05$; **, $padj < 0.05$. Statistical significance from DESeq2 analysis including a combinatorial effect (~genotype+genotype:condition). **(h)** *VND6* expression corresponding single cell sequencing data extracted from the RootCellAtlas (https://rootatlas.netlify.app/rootAtlas) **(i)** Quantification of roots exhibiting protoxylem gaps. Numbers indicate n; letters indicate statistical significance with multiple Fisher's exact test and BH correction, $p < 0.05$.

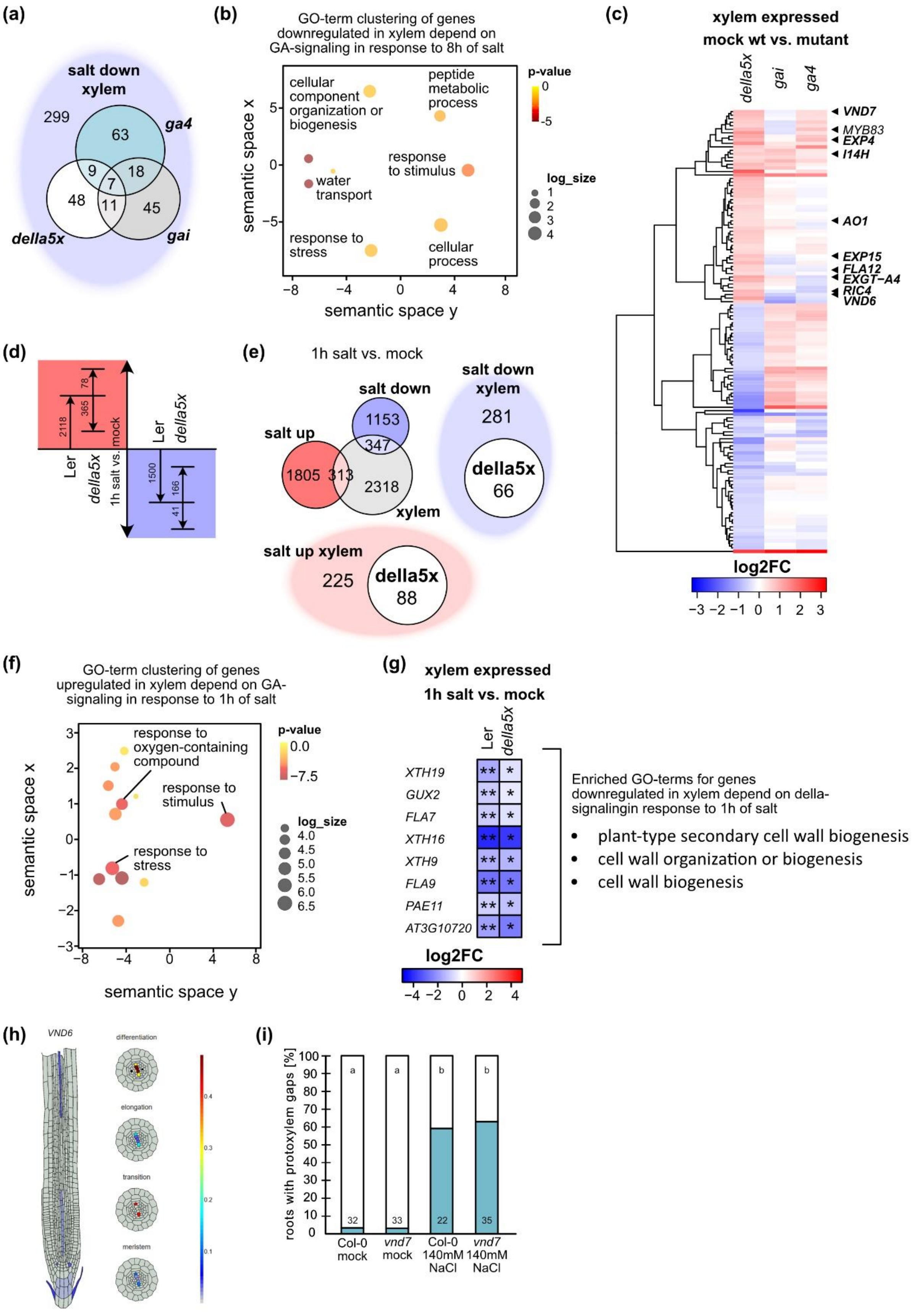

(a)
salt down xylem
299
63
ga4
9
18
7
48
11
45
della5x
gai
(b)
GO-term clustering of genes downregulated in xylem depend on GA-signaling in response to 8h of salt
cellular component organization or biogenesis
peptide metabolic process
water transport
response to stimulus
response to stress
cellular process
p-value
log_size
semantic space x
semantic space y
(c)
xylem expressed
mock wt vs. mutant
della5x
gai
ga4
VND7
MYB83
EXP4
I14H
AO1
EXP15
FLA12
EXGT−A4
RIC4
VND6
log2FC
(d)
Ler
della5x
1h salt vs. mock
2118
365
78
1500
166
41
(e)
1h salt vs. mock
salt down
1153
salt up
347
1805
313
2318
xylem
salt down xylem
281
della5x
66
salt up xylem
225
della5x
88
(f)
GO-term clustering of genes upregulated in xylem depend on GA-signaling in response to 1h of salt
response to oxygen-containing compound
response to stimulus
response to stress
p-value
log_size
semantic space x
semantic space y
(g)
xylem expressed
1h salt vs. mock
Ler
della5x
XTH19
GUX2
FLA7
XTH16
XTH9
FLA9
PAE11
AT3G10720
log2FC
Enriched GO-terms for genes downregulated in xylem depend on della-signalingin response to 1h of salt
• plant-type secondary cell wall biogenesis
• cell wall organization or biogenesis
• cell wall biogenesis
(h)
VND6
differentiation
elongation
transition
meristem
(i)
roots with protoxylem gaps [%]
a
a
b
b
32
33
22
35
Col-0 mock
vnd7 mock
Col-0 140mM NaCl
vnd7 140mM NaCl

Fig. S7 Cell wall modifying enzymes in xylem gap formation. (a) Tissue specific expression of *EXP1* and *XTH20* during a time course of salt exposure extracted from the eFP browser (Geng *et al.*, 2013). **(b)** Heatmap showing expression of alpha-expansins after 8h of salt exposure in L*er* (wild type), *della5x* (*gai-t6 rga-t2 rgl1-1 rgl2-1 rgl3-4*), *gai* and *ga4* *, $p < 0.05$; **, padj < 0.05 (see also Supplementary Table 2). Statistical significance from DESeq2 analysis including a combinatorial effect (~genotype+genotype:condition). **(c)** Quantification of roots exhibiting protoxylem gaps. Numbers indicate n; letters indicate statistical significance with multiple Fisher's exact test and BH correction, $p < 0.05$.

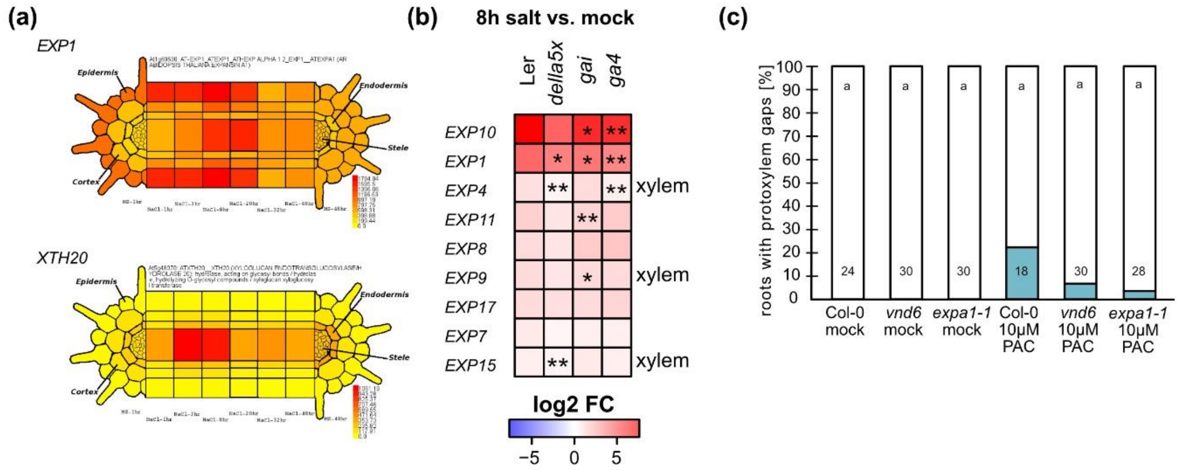

Fig. S8 Enhanced protoxylem gap formation confers increased salt tolerance. (a - c, f, i) Pictures of seedlings of indicated genotypes, grown for 4 days on mock or 200mM NaCl. *della5x* is *gai-t6 rga-t2 rgl1-1 rgl2-1 rgl3-4; vnd1237* is *vnd1 vnd2 vnd3 vnd7*. Arrowheads point at examples of seedlings with green, pale green and white cotyledons. Scale bar, 1 cm. **(d - e)** Quantification of roots exhibiting **(d)** protoxylem or **(e)** outer metaxylem gaps in 6-day old Arabidopsis Col-0 or *vnd1237* grown for 3 days on 140mM NaCl or under mock conditions. (**g**) Quantification of roots exhibiting protoxylem gaps in 6-day old Arabidopsis Col-0 or *ahp6-1* grown for 3 days on 140mM NaCl or under mock conditions. Numbers indicate n; letters indicate statistical significance with multiple Fisher's exact test and BH correction, $p < 0.05$. **(h, j)** Salt tolerance assay after growth on 200mM NaCl or mock conditions for 4 days for 3 day old seedlings. Survival score was calculated by assigning plants with white cotyledons a score of 1, pale green 3 and green 5. These scores were multiplied and then divided by n analyzed plants. Numbers indicate n from **(h)** three or **(j)** four replicates. Horizontal lines indicate median, whiskers indicate 1.5 Interquartile range (IQR), letters indicate statistical significance with Two-way ANOVA, $p < 0.05$. **(k)** Quantification of roots exhibiting protoxylem gaps in 6-day old Arabidopsis Col-0 or L*er* grown for 4 days on 140mM NaCl or under mock conditions. Seedlings with green and white cotyledons were analyzed separately. Numbers indicate n; letters indicate statistical significance with multiple Fisher's exact test and BH correction, $p < 0.05$. **(l)** Salt tolerance assay after growth on 200mM NaCl or mock conditions for 4 days for 7 day old *ahp6-1* seedling. Survival score was calculated by assigning plants with white cotyledons a score of 1, pale green 3 and green 5. These scores were multiplied and then divided by n analyzed plants. Horizontal lines indicate median, whiskers indicate 1.5 Interquartile range (IQR), numbers indicate n from five replicates. Letters indicate statistical significance with Two-way ANOVA, $p < 0.05$.

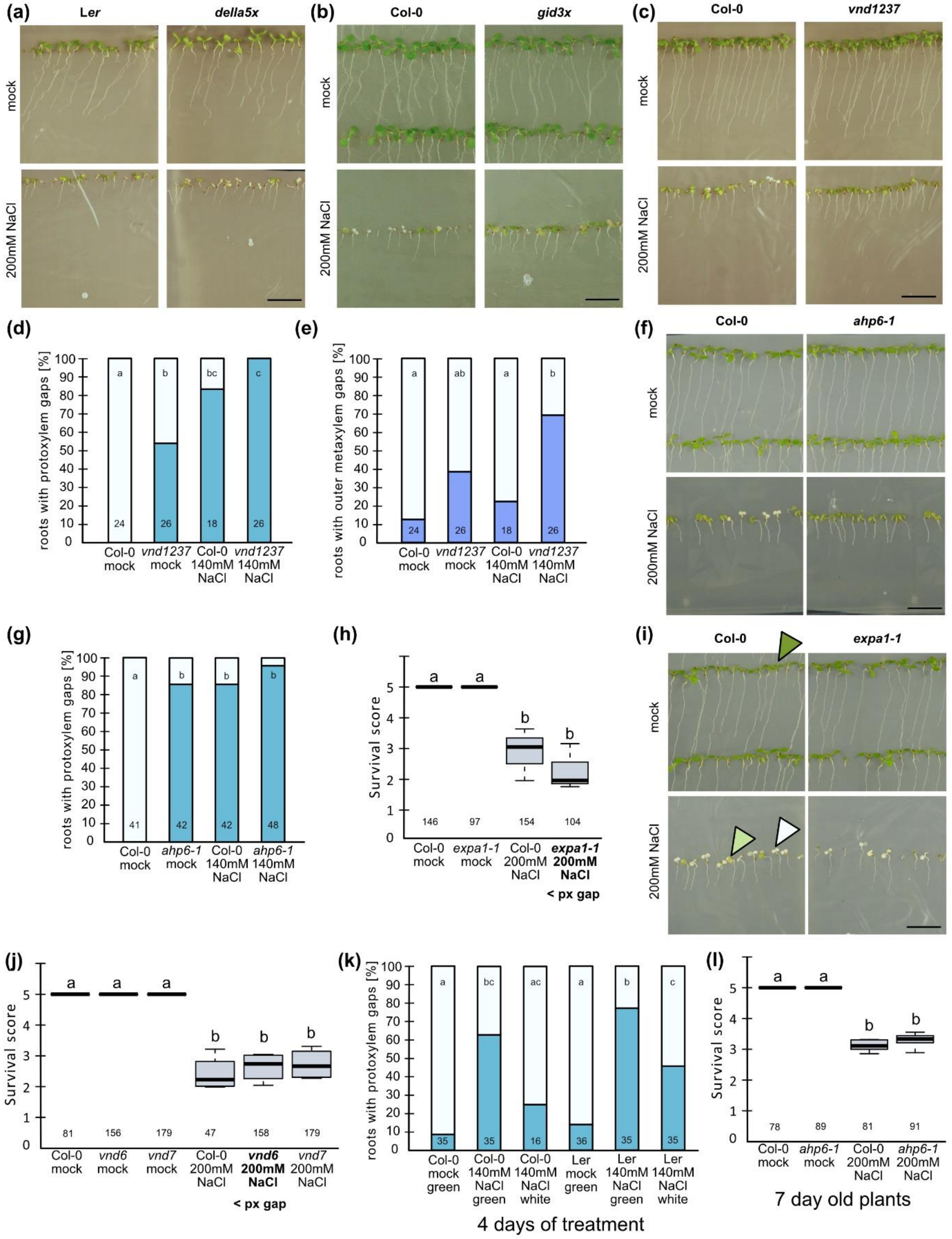
(a) Ler della5x
mock
200mM NaCl
(b) Col-0 gid3x
mock
200mM NaCl
(c) Col-0 vnd1237
mock
200mM NaCl
(d) roots with protoxylem gaps [%]
Col-0 mock
vnd1237 mock
Col-0 140mM NaCl
vnd1237 140mM NaCl
(e) roots with outer metaxylem gaps [%]
Col-0 mock
vnd1237 mock
Col-0 140mM NaCl
vnd1237 140mM NaCl
(f) Col-0 ahp6-1
mock
200mM NaCl
(g) roots with protoxylem gaps [%]
Col-0 mock
ahp6-1 mock
Col-0 140mM NaCl
ahp6-1 140mM NaCl
(h) Survival score
Col-0 mock
expa1-1 mock
Col-0 200mM NaCl
expa1-1 200mM NaCl
< px gap
(i) Col-0 expa1-1
mock
200mM NaCl
(j) Survival score
Col-0 mock
vnd6 mock
vnd7 mock
Col-0 200mM NaCl
vnd6 200mM NaCl
vnd7 200mM NaCl
< px gap
(k) roots with protoxylem gaps [%]
Col-0 mock green
Col-0 140mM NaCl green
Col-0 140mM NaCl white
Ler mock green
Ler 140mM NaCl green
Ler 140mM NaCl white
4 days of treatment
(l) Survival score
Col-0 mock
ahp6-1 mock
Col-0 200mM NaCl
ahp6-1 200mM NaCl
7 day old plants

Table S1 Summary of statistical analyses

Table S2 Differentially expressed genes in roots of L*er*, *della5x*, *gai* and *ga4* upon growth on salt for 1h and 8h, related to Fig. 4, 5, S6, S7. (a) and **(b)** RNA-Seq on roots (1 cm) of L*er* and *della5x* (*gai-t6 rga-t2 rgl1-1 rgl2-1 rgl3*-4) treated for 1h with 140mM NaCl or mock. **(a)** Differential expression analysis evaluating the impact of the mutant background on the effect of 140mM NaCl treatment (combinatorial effect). Log2 fold-changes (log2FC) were extracted from the pairwise comparison of mock-treatment for each genotype, while p values and adjusted p (padj) were extracted from the pairwise comparison between mutant and wildtype. P values and padj for L*er* were extracted from the pairwise comparison with mock treatment. Genes upregulated by NaCl in L*er* with log2FC>0.5 are marked in green lettering, and those with log2FC<-0.5 in red, genes with padj<0.05 are marked in bold, genes with p<0.05 in italics. Genes significantly upregulated upon NaCl in L*er* (padj<0.05), which are significantly reduced in mutant (p<0.05) are marked in blue lettering. **(b)** Differential expression analysis between L*er* and *della5x*. Log2FC, p values and padj were extracted from pairwise comparisons. Genes with log2FC>0.5 in mutant compared to L*er* are marked in green lettering, and those with log2FC<-0.5 in red. Genes with padj<0.05 are marked in bold; genes with p<0.05 in italics. **(c)** and **(d)** RNA-Seq on roots (1 cm) of L*er*, *della5x*, *gai*, *ga4* treated for 8h with 140mM NaCl or mock. **(c)** Differential expression analysis evaluating the impact of the mutant background on the effect of 140mM NaCl treatment (combinatorial effect). Log2FC were extracted from the pairwise comparison of mock-treatment for each genotype, while p values and padj were extracted from the pairwise comparison between mutants and wildtype. P values and padj for L*er* were extracted from the pairwise comparison with mock treatment. Genes upregulated by NaCl in L*er* with log2FC>0.5 are marked in green lettering, and those with log2FC<-0.5 in red, genes with padj<0.05 are marked in bold, genes with p<0.05 in italics. Genes significantly upregulated upon NaCl in L*er* (padj<0.05), which are significantly reduced in mutants (p<0.05) are marked in blue lettering. **(d)** Differential expression analysis between L*er* and *della5x*, *gai*, and/or *ga4*. Log2FC, p values and padj were extracted from pairwise comparisons. Genes with log2FC>0.5 in mutant compared to L*er* are marked in green lettering, and those with log2FC<-0.5 in red. Genes with padj<0.05 are marked in bold; genes with p<0.05 in italics. Xylem_Denyer2019: genes

expressed in the immature xylem identified by scRNASeq (Denyer et al., 2019); Xylem_Wendrich2020: genes expressed in the immature xylem identified by scRNASeq (Wendrich et al., 2020). NA, not available.

Table S3 Enriched GOs from Panther analysis of xylem expressed genes up- and downregulated by 1h/ 8h of salt exposure in DELLA-/ GA-dependent manner

**Xylem expressed genes upregulated by 1h of salt exposure in a DELLA-dependent manner**		
**GO biological process complete**		**P value**
GO:0009737	response to abscisic acid	0.00000836
GO:0033993	response to lipid	0.00000382
GO:0010033	response to organic substance	0.00000935
GO:0042221	response to chemical	4.47E-10
GO:0050896	response to stimulus	3.08E-08
GO:0009725	response to hormone	0.00000468
GO:0009719	response to endogenous stimulus	0.00000678
GO:0097305	response to alcohol	0.00000983
GO:1901700	response to oxygen-containing compound	0.000000119
GO:0009414	response to water deprivation	0.0147
GO:0006950	response to stress	0.000000135
GO:0009415	response to water	0.0182
GO:0001101	response to acid chemical	0.0323
GO:0009628	response to abiotic stimulus	1.18E-09
GO:0006970	response to osmotic stress	0.00355
GO:0009266	response to temperature stimulus	0.0121
**Xylem expressed genes downregulated by 1h of salt exposure in a DELLA-dependent manner**		
**GO biological process complete**		**P value**
GO:0009834	plant-type secondary cell wall biogenesis	0.019
GO:0071554	cell wall organization or biogenesis	0.0108
GO:0042546	cell wall biogenesis	0.0153
**Xylem expressed genes upregulated by 8h of salt exposure in a GA-dependent manner**		
**GO biological process complete**		**P value**
GO:0009414	response to water deprivation	0.000000201
GO:0006950	response to stress	1.34E-10
GO:0050896	response to stimulus	5.31E-10
GO:0009415	response to water	0.00000031
GO:1901700	response to oxygen-containing compound	0.000000389
GO:0042221	response to chemical	2.65E-13
GO:0010035	response to inorganic substance	0.0000297

GO:0001101	response to acid chemical	0.00000102
GO:0009628	response to abiotic stimulus	0.0000137
GO:0009636	response to toxic substance	0.0284
GO:0009737	response to abscisic acid	0.000000889
GO:0033993	response to lipid	0.0000164
GO:0010033	response to organic substance	0.000144
GO:0009725	response to hormone	0.0000284
GO:0009719	response to endogenous stimulus	0.0000456
GO:0097305	response to alcohol	0.00000112
GO:0071669	plant-type cell wall organization or biogenesis	0.0126
GO:0071554	cell wall organization or biogenesis	0.00552
GO:0006970	response to osmotic stress	0.00167
GO:0009605	response to external stimulus	0.000128
**Xylem expressed genes downregulated by 8h of salt exposure in a GA-dependent manner**		
**GO biological process complete**		**P value**
GO:0080170	hydrogen peroxide transmembrane transport	0.0413
GO:0009987	cellular process	0.0139
GO:0006833	water transport	0.00000408
GO:0042044	fluid transport	0.00000408
GO:0006412	translation	0.0297
GO:0043043	peptide biosynthetic process	0.0328
GO:0006518	peptide metabolic process	0.0109
GO:0071840	cellular component organization or biogenesis	0.035
GO:0006950	response to stress	0.0241
GO:0050896	response to stimulus	0.000956

Methods S1 Key resources used in this study

REAGENT or RESOURCE	SOURCE	IDENTIFIER
**Chemicals, peptides, and recombinant proteins**		
Murashige and Skoog Medium (MS)	Duchefa Biochemie	Cat#M0222.0050
MES monohydrate	Duchefa Biochemie	Cat#M1503.0250
Bactoagar	Swab	Cat#B1000-1
Abscisic acid (ABA)	Sigma	Cat#14375-45-2
Gibberellic acid 3	Duchefa Biochemie	Cat#G0907
Gibberellic acid 4+7	Duchefa Biochemie	Cat#G0938
Paclobutrazol (PAC)	Sigma	Cat#46046
Mannitol	VWR Chemicals	Cat#25311.366
Chloralhydrate	Sigma	Cat#15307
Urea	Sigma	Cat#57-13-6
Sodium deoxycholate	Sigma	Cat#1065040250
Xylitol	Sigma	Cat#X3375
Propidium iodide	Sigma	Cat#P4170
**Critical commercial assays**		
RNeasy Plant Mini Kit	Qiagen	Cat#74904
Qubit BR RNA Assay	Invitrogen	Cat#Q10211
**Deposited data**		
Raw and processed RNAseq data files	This study	GEO: GSE195912
Raw and processed RNAseq data files	(Ramachandran *et al.*, 2021)	GEO: GSE169367
**Experimental models: organisms/strains**		
Arabidopsis thaliana: Col-0	Widely distributed	N/A

Arabidopsis thaliana: L*er*	Widely distributed	N/A
Solanum lycopersicum cv. Moneymaker	Nelson garden and Plantagen	N/A
Beta vulgaris cv. Davinci	Maribo Hilleshög/ Christina Dixelius, SLU	N/A
Eutrema salsugineum	Claudia Köhler, Max Planck Inst. Potsdam	N/A
Arabidopsis thaliana: *abi1-1C* in Col-0 background	(Kanno *et al.*, 2012)	N/A
Arabidopsis thaliana: snrk2.2/2.3 in Col-0 background		
Arabidopsis thaliana: *vnd1 vnd2 vnd3 vnd7* in Col-0 background	(Ramachandran *et al.*, 2021)	N/A
Arabidopsis thaliana: gai-t6 rga-t2 rgl1-1 rgl2-1 rgl3-4 in L*er* background	(Koini *et al.*, 2009)	N/A
Arabidopsis thaliana: gai-t6 rga-t2 rgl1-1 rgl2-1 in L*er* background	(Cheng *et al.*, 2004)	N/A
Arabidopsis thaliana: rga-28 in Col-0 background	(Tyler *et al.*, 2004)	N/A
Arabidopsis thaliana: rgl-3 in Col-0 background	NASC seed stock center	N/A
Arabidopsis thaliana: rga-28 rgl3-5 in Col-0 background	This study	N/A
Arabidopsis thaliana: gai-td1 in Col-0 background	(Plackett *et al.*, 2014)	N/A
Arabidopsis thaliana: gai in L*er* background	(Koorneef *et al.*, 1985)	N/A
Arabidopsis thaliana: gid1a-2 gid1b-3 gid1c-1 in Col-0 background	(Griffiths *et al.*, 2006)	N/A
Arabidopsis thaliana: ga1-3 in L*er* background	(Sun *et al.*, 1992)	N/A
Arabidopsis thaliana: ga1-5 in L*er* background	(Sun *et al.*, 1992)	N/A

Arabidopsis thaliana: ga4 in *Ler* background	(Koornneef & van der Veen, 1980; Talon *et al.*, 1990)	N/A
Arabidopsis thaliana: ahp6-1 in Col-0 background	(Mähönen *et al.*, 2006)	N/A
Arabidopsis thaliana: expa1-1 in Col-0 background	NASC seed stock center	N/A
Arabidopsis thaliana: *vnd6* in Col-0 background	(Kubo *et al.*, 2005)	N/A
Arabidopsis thaliana: pANT:his-YFP in Col-0 background	(Randall *et al.*, 2015)	N/A
Arabidopsis thaliana: pVND7:YFP-NLS in Col-0 background	(Kubo *et al.*, 2005)	N/A
Arabidopsis thaliana: RGA:GFP-RGA in *Ler* background	(Silverstone *et al.*, 2001)	N/A
**Software and algorithms**		
Zeiss Zen Black 2.3 SP1	Zeiss	https://www.zeiss.com/
Zeiss Zen Blue 2.3 lite and 2.5	Zeiss	https://www.zeiss.com/
R 4.02 and R studio 1.2.5019	(R Core Team; RStudioTeam, 2019)	https://www.r-project.org/ https://rstudio.com/
Microsoft Excel 2016	Microsoft	N/A
CellSet v1.5.1	(Pound *et al.*, 2012)	https://sourceforge.net/projects/cellset/
Affinity Designer 1.7	Affinity	N/A
Fiji/Image J 2.0.0 Win64 or 2.0.0-rc-68/1.52h	(Schindelin *et al.*, 2012)	https://fiji.sc/

Bioconductor 3.11	Bioconductor (Huber *et al.*, 2015)	https://bioconductor.org/
Fastp	(Chen *et al.*, 2018)	https://github.com/OpenGene/fastp
MulitQC	(Ewels *et al.*, 2016)	https://multiqc.info/
SortMeRNA	(Kopylova *et al.*, 2012)	https://bioinfo.lifl.fr/RNA/sortmerna/
Trimmomatic	(Bolger *et al.*, 2014)	http://www.usadellab.org/cms/?page=trimmomatic
FastQC	(Andrews, 2010)	https://www.bioinformatics.babraham.ac.uk/projects/fastqc/
HiSAT2	(Kim *et al.*, 2019)	http://daehwankimlab.github.io/hisat2/
HTSeq_Count	(Anders *et al.*, 2015)	https://htseq.readthedocs.io/en/release_0.9.1/count.html
PANTHER 16.0	(Mi *et al.*, 2019, 2021)	http://go.pantherdb.org/
REVIGO	(Supek *et al.*, 2011)	http://revigo.irb.hr/
**Other**		
Zeiss LSM780 confocal microscope	Zeiss	https://www.zeiss.com/
Zeiss LSM800 confocal microscope	Zeiss	https://www.zeiss.com/

Zeiss Axioscope A1	Zeiss	https://www.zeiss.com/

References

Anders S, Pyl PT, Huber W. **2015**. HTSeq-A Python framework to work with high-throughput sequencing data. *Bioinformatics* **31**: 166–169.

Andrews S. **2010**. FASTQC. A quality control tool for high throughput sequence data.

Bolger AM, Lohse M, Usadel B. **2014**. Trimmomatic: A flexible trimmer for Illumina sequence data. *Bioinformatics* **30**: 2114–2120.

Chen S, Zhou Y, Chen Y, Gu J. **2018**. Fastp: An ultra-fast all-in-one FASTQ preprocessor. *Bioinformatics* **34**: i884–i890.

Cheng H, Qin L, Lee S, Fu X, Richards DE, Cao D, Luo D, Harberd NP, Peng J. **2004**. Gibberellin regulates Arabidopsis floral development via suppression of DELLA protein function. *Development* **131**: 1055–1064.

Ewels P, Magnusson M, Lundin S, Käller M. **2016**. MultiQC: Summarize analysis results for multiple tools and samples in a single report. *Bioinformatics* **32**: 3047–3048.

Geng Y, Wu R, Wee CW, Xie F, Wei X, Chan PMY, Tham C, Duan L, Dinneny JR. **2013**. A spatio-temporal understanding of growth regulation during the salt stress response in Arabidopsis. *Plant Cell* **25**: 2132–2154.

Griffiths J, Murase K, Rieu I, Zentella R, Zhang ZL, Powers SJ, Gong F, Phillips AL, Hedden P, Sun TP, *et al.* **2006**. Genetic characterization and functional analysis of the GID1 gibberellin receptors in Arabidopsis. *Plant Cell* **18**: 3399–3414.

Huber W, Carey VJ, Gentleman R, Anders S, Carlson M, Carvalho BS, Bravo HC, Davis

S, Gatto L, Girke T, *et al.* 2015. Orchestrating high-throughput genomic analysis with Bioconductor. *Nature Methods* **12**: 115–121.

Kanno Y, Hanada A, Chiba Y, Ichikawa T, Nakazawa M, Matsui M, Koshiba T, Kamiya Y, Seo M. **2012**. Identification of an abscisic acid transporter by functional screening using the receptor complex as a sensor. *Proceedings of the National Academy of Sciences* **109**: 9653–9658.

Kim D, Paggi JM, Park C, Bennett C, Salzberg SL. **2019**. Graph-based genome alignment and genotyping with HISAT2 and HISAT-genotype. *Nature Biotechnology* **37**: 907–915.

Koini MA, Alvey L, Allen T, Tilley CA, Harberd NP, Whitelam GC, Franklin KA. **2009**. High Temperature-Mediated Adaptations in Plant Architecture Require the bHLH Transcription Factor PIF4. *Current Biology* **19**: 408–413.

Koorneef M, Elgersma A, Hanhart CJ, van Loenen-Martinet EP, van Rijn L, Zeevaart JAD. **1985**. A gibberellin insensitive mutant of *Arabidopsis thaliana*. *Physiologia Plantarum* **65**: 33–39.

Koornneef M, van der Veen JH. **1980**. Induction and analysis of gibberellin sensitive mutants in *Arabidopsis thaliana* (L.) heynh. *Theoretical and Applied Genetics* **58**: 257–263.

Kopylova E, Noé L, Touzet H. **2012**. SortMeRNA: Fast and accurate filtering of ribosomal RNAs in metatranscriptomic data. *Bioinformatics* **28**: 3211–3217.

Kubo M, Udagawa M, Nishikubo N, Horiguchi G, Yamaguchi M, Ito J, Mimura T, Fukuda H, Demura T. **2005**. Transcription switches for protoxylem and metaxylem vessel formation. *Genes and Development* **19**: 1855–1860.

Mähönen AP, Bishopp A, Higuchi M, Nieminen KM, Kinoshita K, Törmäkangas K, Ikeda Y, Oka A, Kakimoto T, Helariutta Y. **2006**. Cytokinin Signaling and Its Inhibitor AHP6 Regulate Cell Fate During Vascular Development. *Science* **311**: 94–98.

Mi H, Ebert D, Muruganujan A, Mills C, Albou LP, Mushayamaha T, Thomas PD. **2021**. PANTHER version 16: A revised family classification, tree-based classification tool, enhancer regions and extensive API. *Nucleic Acids Research* **49**: D394–D403.

Mi H, Muruganujan A, Huang X, Ebert D, Mills C, Guo X, Thomas PD. **2019**. Protocol Update for large-scale genome and gene function analysis with the PANTHER classification system (v.14.0). *Nature Protocols* **14**: 703–721.

Plackett ARG, Ferguson AC, Powers SJ, Wanchoo-Kohli A, Phillips AL, Wilson ZA, Hedden P, Thomas SG. **2014**. DELLA activity is required for successful pollen development in the Columbia ecotype of Arabidopsis. *New Phytologist* **201**: 825–836.

Pound MP, French AP, Wells DM, Bennett MJ, Pridmorea TP. **2012**. CellSeT: Novel software to extract and analyze structured networks of plant cells from confocal images. *Plant Cell* **24**: 1353–1361.

R Core Team. R: A language and environment for statistical computing. *R Foundation for Statistical Computing, Vienna, Austria.*

Ramachandran P, Augstein F, Mazumdar S, Nguyen T Van, Minina EA, Melnyk CW, Carlsbecker A. **2021**. Abscisic acid signaling activates distinct VND transcription factors to promote xylem differentiation in Arabidopsis. *Current Biology* **31**: 1–9.

Randall RS, Miyashima S, Blomster T, Zhang J, Elo A, Karlberg A, Immanen J, Nieminen K, Lee JY, Kakimoto T, *et al.* 2015. AINTEGUMENTA and the D-type cyclin CYCD3;1 regulate root secondary growth and respond to cytokinins. *Biology Open* **4**: 1229–1236.

RStudioTeam. **2019**. RStudio: Integrated Development for R. *RStudio, Inc., Boston*.

Schindelin J, Arganda-Carreras I, Frise E, Kaynig V, Longair M, Pietzsch T, Preibisch S, Rueden C, Saalfeld S, Schmid B, *et al.* 2012. Fiji: An open-source platform for biological-

image analysis. *Nature Methods* **9**: 676–682.

Silverstone AL, Jung H-S, Dill A, Kawaide H, Kamiya Y, Sun T. **2001**. Repressing a Repressor: Gibberellin-Induced Rapid Reduction of the RGA Protein in Arabidopsis. *The Plant Cell* **13**: 1555–1566.

Sun T, Goodman HM, Ausubel FM. **1992**. Cloning the Arabidopsis *GA1* Locus by Genomic Subtraction. *The Plant Cell* **4**: 119–128.

Supek F, Bošnjak M, Škunca N, Šmuc T. **2011**. Revigo summarizes and visualizes long lists of gene ontology terms. *PLoS ONE* **6**.

Talon M, Koornneef M, Zeevaart JAD. **1990**. Endogenous gibberellins in *Arabidopsis thaliana* and possible steps blocked in the biosynthetic pathways of the semidwarf *ga4* and *ga5* mutants. *Proceedings of the National Academy of Sciences of the United States of America* **87**: 7983–7987.

Tyler L, Thomas SG, Hu J, Dill A, Alonso JM, Ecker JR, Sun TP. **2004**. Della proteins and gibberellin-regulated seed germination and floral development in Arabidopsis. *Plant Physiology* **135**: 1008–1019.
